# Identification and validation of risk loci for osteochondrosis in standardbreds

**DOI:** 10.1186/s12864-016-2385-z

**Published:** 2016-01-12

**Authors:** Annette M. McCoy, Samantha K. Beeson, Rebecca K. Splan, Sigrid Lykkjen, Sarah L. Ralston, James R. Mickelson, Molly E McCue

**Affiliations:** Veterinary Population Medicine Department, University of Minnesota, 1365 Gortner Ave., St. Paul, MN USA; Department of Veterinary Clinical Medicine, University of Illinois, 1008 Hazelwood Dr., Urbana, IL USA; Department of Animal and Poultry Sciences, Virginia Tech, 3470 Litton Reaves Hall, Blacksburg, VA USA; Faculty of Veterinary Medicine and Biosciences, Norwegian University of Life Sciences, NMBU-School of Veterinary Science, P.O. Box 8146 Dep., Oslo, Norway; School of Environmental and Biological Sciences, Rutgers, The State University of New Jersey, 84 Lipman Dr., New Brunswick, NJ USA; Veterinary Biological Sciences Department, University of Minnesota, 1988 Fitch Ave., St. Paul, MN USA

**Keywords:** Developmental orthopedic disease, Cartilage, Genetic risk, Horse, Genome-wide association analysis

## Abstract

**Background:**

Osteochondrosis (OC), simply defined as a failure of endochondral ossification, is a complex disease with both genetic and environmental risk factors that is commonly diagnosed in young horses, as well as other domestic species. Although up to 50 % of the risk for developing OC is reportedly inherited, specific genes and alleles underlying risk are thus far completely unknown. Regions of the genome identified as associated with OC vary across studies in different populations of horses. In this study, we used a cohort of Standardbred horses from the U.S. (*n* = 182) specifically selected for a shared early environment (to reduce confounding factors) to identify regions of the genome associated with tarsal OC. Subsequently, putative risk variants within these regions were evaluated in both the discovery population and an independently sampled validation population of Norwegian Standardbreds (*n* = 139) with tarsal OC.

**Results:**

After genome-wide association analysis of imputed data with information from >200,000 single nucleotide polymorphisms, two regions on equine chromosome 14 were associated with OC in the discovery cohort. Variant discovery in these and 30 additional regions of interest (including 11 from other published studies) was performed via whole-genome sequencing. 240 putative risk variants from 10 chromosomes were subsequently genotyped in both the discovery and validation cohorts. After correction for population structure, gait (trot or pace) and sex, the variants most highly associated with OC status in both populations were located within the chromosome 14 regions of association.

**Conclusions:**

The association of putative risk alleles from within the same regions with disease status in two independent populations of Standardbreds suggest that these are true risk loci in this breed, although population-specific risk factors may still exist. Evaluation of these loci in other populations will help determine if they are specific to the Standardbred breed, or to tarsal OC or are universal risk loci for OC. Further work is needed to identify the specific variants underlying OC risk within these loci. This is the first step towards the long-term goal of constructing a genetic risk model for OC that allows for genetic testing and quantification of risk in individuals.

**Electronic supplementary material:**

The online version of this article (doi:10.1186/s12864-016-2385-z) contains supplementary material, which is available to authorized users.

## Background

Osteochondrosis (OC) is a commonly diagnosed developmental orthopedic disease that is defined as a focal failure of endochondral ossification, the process by which a cartilage template becomes bone in the limbs of a growing animal [1]. It is characterized by the presence of abnormal cartilage within a joint that may be thickened, soft or collapsed or separated entirely from the underlying bone. In the last case, the condition is commonly referred to as osteochondrosis dissecans (OCD) [2]. OC is widely recognized in young horses across breeds (as well as many other species) and is of particular interest because of its potential to cause joint effusion and/or lameness in horses preparing for sales and entering training.

It has been postulated that OC could be caused by either abnormal forces on normal cartilage or by normal forces on abnormal cartilage [3], but the exact pathophysiology is not yet understood. Evidence from naturally-occurring disease and experimental models suggests that abnormalities in vascular supply to the articular cartilage and subchondral bone at anatomical predilection sites (i.e. within the tarsus, stifle and fetlock) underlie the condition [4–6]. However, alternative theories include abnormal extracellular matrix maturation and inherited endoplasmic reticulum storage disorders [7–9]. Additional contributing factors that have been suggested include nutrition, exercise, conformation and other biomechanical factors, trauma, stress response, *in utero* environment and hormonal interactions [10–12]. While it is generally accepted that a combination of genetic and environmental factors influence the development of lesions, response to environmental management alone is limited, highlighting the importance of genetics in this disease [13–16].

The genetic contribution of OC risk has been quantified in a limited number of breeds considered particularly prone to the condition, including Standardbreds [17–19], French Trotters [20], Warmbloods [21–23], and South German Coldbloods [24]. Based on these reports, it can be estimated that between 15 and 52 % of the global risk for developing OC can be attributed to genetic factors. Variation in heritability estimates between populations is to be expected for any trait due to differences in population history, gene frequency and environmental exposures [25]. This is particularly true for OC since it has known environmental interactions and likely has multiple genetic alleles conferring susceptibility.

The presence of OC across domestic horse populations, including a feral horse population [26], as well as shared major anatomical predilection sites and lesion morphology [27], suggests a common underlying pathophysiology and shared major genetic risk factors across breeds. However, to date, the specific genes and alleles underlying OC risk in the horse are completely unknown. Genome-wide association studies (GWAS) have been performed in a number of breeds using both microsatellite markers and single nucleotide polymorphism (SNP) beadchips (summarized in [28]). These studies and follow-up fine-mapping efforts [29, 30] have identified multiple chromosomal loci that could potentially contribute to the heritability of OC. However, the findings have not been consistent across studies, and only a single attempt to validate putative risk loci in a second population has been reported [31]. Further, while many potential candidate genes have been identified, only one has been investigated [32], and a functional allele conferring risk was not identified. The lack of agreement in previous mapping studies may reflect confounding due to environmental risk factors and variability in phenotypic criteria for OC, or may represent true population differences in risk alleles. Selection of a study cohort made up of related individuals with a shared early environment who all exhibit a similar phenotype may help to address these potential limitations of previous GWAS.

Here we describe the discovery of putative functional variants and validation of risk loci underlying genetic risk for tarsal OC in Standardbreds. Chromosomal regions of interest were identified by genome-wide association, enhanced by genotype imputation to a high SNP density, in a discovery cohort of related Standardbred yearlings born and raised on a single breeding farm in the United States. Whole-genome sequencing was performed for a subset of these individuals for the purposes of variant discovery. Variants were then prioritized based on predicted functional effect and segregation with OC status. Selected putative functional variants were genotyped in both the discovery population and an independent validation cohort of Norwegian Standardbreds with tarsal OC. This is the first report of specific putative risk alleles associated with disease in two independent populations, and is an initial step towards the long-term goal of developing a genetic risk model for OC that would allow for genetic testing and quantification of risk in individual horses.

## Results

### Genome-Wide Association (GWA) analysis

**Fig. 1:**
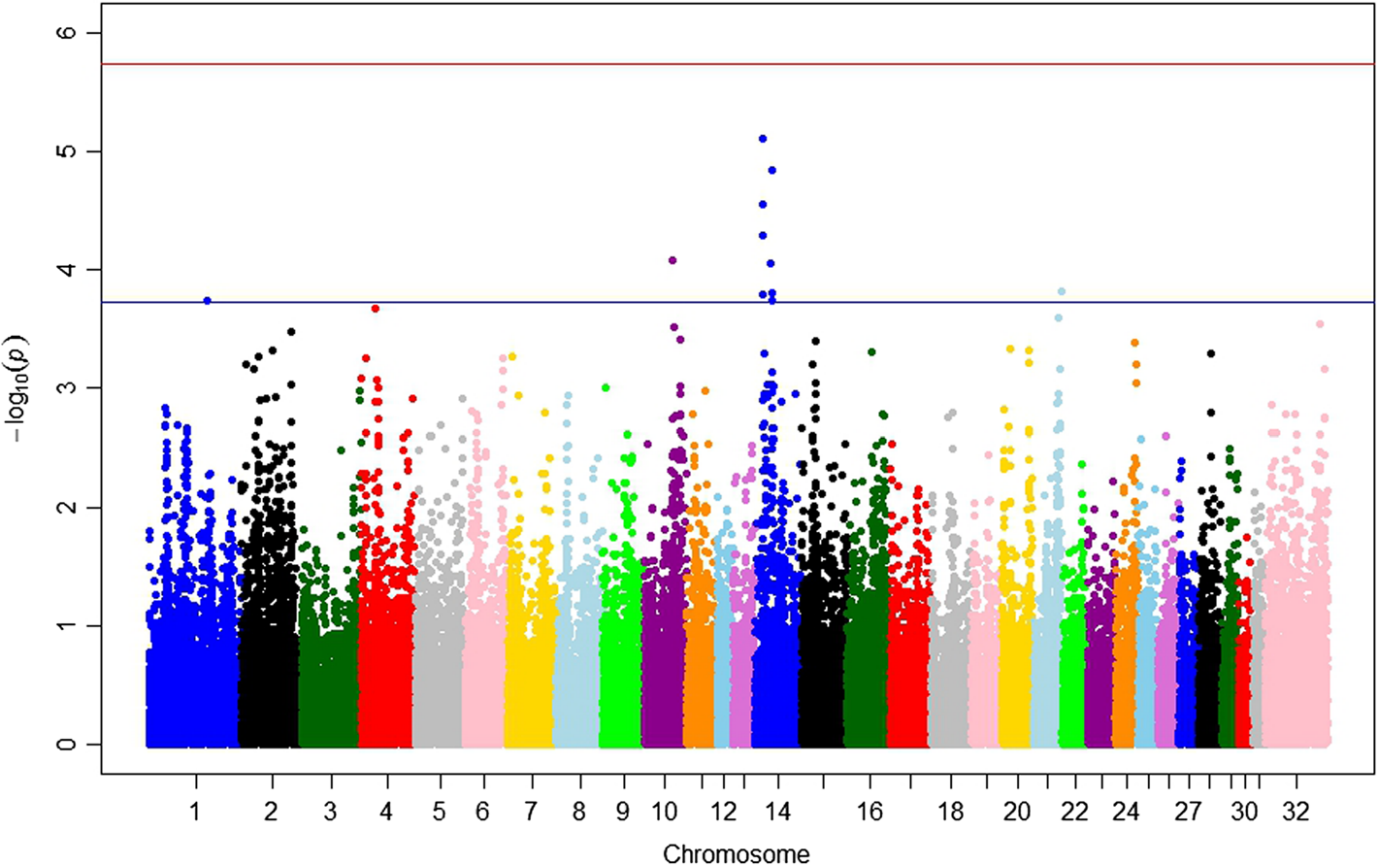
Manhattan plot of results from mixed model analysis using GEMMA. The 31 autosomal and X chromosome (32) are represented in different colors along the x-axis and the –log (*p*-value) is on the y-axis. Each colored dot represents a SNP. Top hits are on ECA14. See Table 1 for specific SNPs and *p*-values. The red horizontal line represents the level of genome-wide significance (p < 1.86 x 10^-6^); the blue line represents the cutoff for moderate association (p < 1.86 x 10^-4^)

Horses in the discovery cohort (*n* = 182; 70 OC-affected, 112 unaffected) were genotyped on either the first- (Illumina Equine SNP50) or second-generation (Illumina Equine SNP70) equine SNP chip. In order to combine data from the two platforms without loss of marker information, genotype imputation was performed using a previously validated pipeline [33]. 61,046 markers were available for GWA analysis. The mixed model analysis in GEMMA, including sex and gait (pace or trot) as covariates, revealed 12 SNPs, nine on ECA14 and one each on ECA1, 10 and 21 that showed moderate evidence of association with OC status (*p* ≤ 1.86 × 10^−4^ as determined by the likelihood ratio test) (Fig. 1 and Table 1). The nine SNPs on ECA14 defined two distinct loci. Five SNPs were loosely clustered between ~16.4 and 17.8 Mb (with a slightly less significant hit at ~18.3 Mb), while four SNPs defined a second region of interest between ~33.6 and 36.2 Mb. Forty-two named genes, 13 predicted pseudogenes and 3 non-coding RNAs were located within the two regions of interest on ECA14 (Additional file 1: Table S1).

**Table 1:** Top GWA SNPs from GEMMA mixed model analysis

Chr	bp	*P*-value likelihood ratio test
**14**	**16401778**	7.99E-06
**14**	**34284113**	1.43E-05
**14**	**17858976**	2.77E-05
**14**	**17866794**	2.77E-05
**14**	**17626659**	5.17E-05
**10**	**56558910**	8.32E-05
**14**	**33630011**	8.91E-05
**21**	**54501469**	1.51E-04
**14**	**34366588**	1.55E-04
**14**	**17534553**	1.64E-04
**1**	**118288481**	1.80E-04
**14**	**36214363**	1.83E-04
4	28769871	2.10E-04
21	48322513	2.51E-04
32	108097077	2.88E-04
10	58040174	3.07E-04
2	99965882	3.32E-04
10	72307543	3.89E-04
15	28682409	4.01E-04
24	38866310	4.13E-04
20	16930188	4.72E-04
2	61394335	4.78E-04
20	55342664	4.85E-04
16	50579676	4.93E-04
16	50839833	4.93E-04
16	51040831	4.93E-04
16	51098759	4.93E-04
28	26310499	5.01E-04
14	18305845	5.16E-04

SNPs in bold are moderately associated with disease (1.86 × 10^−6^ < *p* < 1.86 × 10^−4^)

*Chr* chromosome, *bp* base pair

Haplotype analysis of the SNPs included in the GWA was performed within the two regions of association identified on ECA14. Within the region from ~16.4 to 18.3 Mb, nine haplotype blocks were identified, made up of 2–8 SNPs and ranging in size from 8 bp to ~230 kb. Haplotypes within three of these blocks were significantly associated with OC (permuted *p*-value <0.05); a haplotype in a fourth block was moderately associated with OC (permuted *p*-value 0.08). The overall frequency of the three significantly associated haplotypes ranged from 13.4 to 15.2 %, and they were more common in controls (17.8- 20.1 %) than cases (6.4–7.2 %). Within the region from ~33.6 to 36.2 Mb, 22 haplotype blocks were identified, made up of 2–12 SNPs and ranging in size from 4.6 kb to ~484 kb. A haplotype from a single block was significantly associated with OC (permuted *p*-value 0.033), and haplotypes from two additional blocks were moderately associated with OC (permuted *p*-values 0.12–0.129). The frequency of the significantly associated haplotype was 14.9 %, similar to the haplotypes in the other region; however, it was more common in cases (22.5 %) than controls (10.3 %). The two moderately associated haplotypes from this region were present less frequently in the population (5.8–6.9 %), but were also more common in cases (10.1–11.6 %) than controls (3.1–4 %) (Additional file 1: Table S2).

Two additional GWA analyses were performed in the discovery cohort (*n* = 182) after imputation enabled by the large SNP lists generated during development of the new commercial equine SNP chip (see Methods). After pruning in GEMMA, 123,352 and 243,115 markers from the 670 k and 2 M sets, respectively, were available for inclusion in the GWA analysis. Sex and gait were included as covariates in the GEMMA mixed model. Analysis of the 670 k set revealed 10 SNPs that showed moderate evidence of association with OC status (*p* ≤ 3.3 × 10^−5^ as determined by the likelihood ratio test). An additional 39 SNPs on 15 chromosomes were within one order of magnitude of this significance. Analysis of the 2 M set revealed 30 SNPs that showed moderate evidence of association with OC status (*p* ≤ 4.8 × 10^−5^ as determined by the likelihood ratio test). An additional 66 SNPs on 16 chromosomes were within one order of magnitude of this significance. The most significantly associated SNPs in both analyses were all located on ECA14, within the regions of interest defined by the original GWA analysis (Additional file 1: Table S3).

### Whole-genome sequencing

Twelve individuals (6 affected, 6 unaffected) were sequenced for a target of 6x coverage depth (actual coverage 4.7x to 7.9x; mean 6.4x). Six individuals (3 affected, 3 unaffected) were sequenced for a target of 12x coverage depth (actual coverage 10x to 13.1x; mean 12.2x). After filtering, 14,588,812 variants were called, at an average of 1 variant every 162 base pairs. Of these, 13,157,608 were SNPs, 671,144 were insertions and 760,060 were deletions. The vast majority of variants, over 14 million (99.1 %), were not predicted to have any functional effect. Of the 152,700 variants predicted to have some functional effect, 85,916 were of low impact (mostly synonymous SNPs), 57,122 were of moderate impact, and 9,662 were of high impact (Additional file 1: Table S4).

### Sequenom genotyping in the discovery cohort

Approximately 1.5 million variants were evaluated from a total of 32 chromosomal regions that were either identified as regions of interest in our GWAS or were chromosomal regions previously reported to be associated with tarsal OC (see Background and Additional file 1: Table S5). Alleles were prioritized for follow-up according to predicted functional effect and segregation with OC status in the sequenced cohort. A high-throughput Sequenom genotyping assay was selected as the most efficient way to evaluate a large number of prioritized alleles for association with OC in a larger cohort. In total, 240 variants on 10 chromosomes were genotyped in 180 horses from the discovery population (Additional file 1: Table S6). An additional 98 ancestry informative markers (AIMs) were genotyped to use in construction of a mixed model analysis to control for population structure [34]. Genomic heritability of OC, based on these markers, was estimated to be 0.19 ± 0.38 (*p* < 0.001) on the binomial scale, or 0.10 on the underlying normal scale [35].

After pruning in GEMMA, 164 SNPs were available for analysis. Sex and gait were included as covariates in the model. The most significantly associated SNP (*p* = 4 × 10^−4^) was located in the first intron of *ARHGAP26* (Rho GTPase activating protein 26) on ECA14 (chr14.34391965). The alternate allele for this SNP was found in 20 % of cases and 8 % of controls. Of the 23 variants with uncorrected *p*-values < 0.05, ten (*p* = 0.022–4 × 10^−4^) were located within or immediately adjacent to the two ECA14 regions of interest identified in the GWAS (Table 2). Although Sequenom SNPs were not chosen with regard to their location relative to haplotypes in the GWA data, four of these ten ECA14 SNPs (chr14.34391965, chr14.18029925, chr14.18034557, chr14.18059791) were located within the boundaries of haplotypes that were significantly or moderately associated with OC (see *Genome-Wide Association (GWA) analysis*).

**Table 2:** Top SNPs from GEMMA mixed model analysis of Sequenom genotyping data in the discovery cohort

Chr	bp	*P*-value likelihood ratio test	Annotation
14	34391965	4.00E-04	Intron ARHGAP26
14	35363931	1.24E-03	Intron FCHSD1
14	16854653	2.23E-03	Synonymous exon 5 CCNG1
14	34803961	2.25E-03	Downstream SPRY4
14	37127327	2.25E-03	Downstream UBE2D2
10	57350466	2.28E-03	Intron PDSS2
14	18034557	5.97E-03	Intron GABRA1
10	55605051	6.27E-03	Downstream PREP
14	18059791	6.96E-03	Intron GABRA1
21	50348105	7.37E-03	Intron SEMA5A
14	16782922	9.55E-03	Synonymous exon 1 MAT2B
21	53443537	9.69E-03	Downstream ADAMTS1
14	18029925	1.13E-02	Synonymous exon 8 GABRA1
2	99999249	1.24E-02	Intergenic
21	48664783	2.20E-02	Intron CTNND2
14	18528304	2.22E-02	Intron GABRB2
10	57303131	2.23E-02	Intron PDSS2
21	50383063	2.58E-02	Intron SEMA5A
16	14358731	2.69E-02	Nonsynonymous exon 8 CNTN6
10	56817838	2.92E-02	Intron QRSL1
21	49882816	3.39E-02	Synonymous exon 1 TAS2R1
1	118771557	3.64E-02	Intron PPCDC
1	118401125	4.42E-02	Downstream SNUPN

*Chr* chromosome, *bp* base pair

### Sequenom genotyping in the validation cohort

Horses in the validation cohort (*n* = 139; 60 OC-affected, 79 unaffected) were genotyped using the same custom Sequenom assay as the discovery cohort. After pruning in GEMMA, 176 SNPs were available for analysis. As all horses in this cohort were trotters, only sex was included as a covariate in the model. The most significantly associated SNP (*p* = 0.0014) was a missense mutation in exon 9 of *GABRA6* (gamma-aminobutyric acid (GABA) A receptor, alpha 6) on ECA14 (chr14.18198820). The alternate allele for this SNP was found in 40 % of cases and 23 % of controls, and it was located on the edge of a haplotype found to be significantly associated with OC in the GWA data from the discovery cohort (see *Genome-Wide Association (GWA) Analysis*). Of the 14 variants with uncorrected *p*-values < 0.05, four (*p* = 0.049–0.0014) were from the ECA14 regions of interest identified in the discovery cohort GWAS. Six variants were from regions reported to be associated with OC in the published GWAS for the validation cohort (ECA1 [*n* = 3], ECA3 [*n* = 1], and ECA5 [*n* = 2]) (Table 3) [36]. Interestingly, for all but one of these six SNPs, the alternate allele was more frequent in the unaffected horses (range 17–54 %) than in the affected horses (range 8–33 %). In contrast, the alternate allele for all four of the most significant ECA14 SNPs was more common in the affected individuals (range 32–40 % vs. 16–23 % in unaffected) (Additional file 1: Table S7). SNPs with *p* < 0.05 from two other chromosomal regions were shared between the two populations: ECA10 (~55.6–57.3 Mb) and ECA21 (~48.6–53.4 Mb). These regions of interest were identified from the discovery population GWAS, although they were less significantly associated with OC than the regions on ECA14 (Table 1).

**Table 3:** Top SNPs from GEMMA mixed model analysis of Sequenom genotyping data in the validation cohort

Chr	bp	*P*-value likelihood ratio test	Annotation
14	18198820	1.41E-03	Nonsynonymous exon 9 GABRA6
3	107352236	1.57E-03	Nonsynonymous exon 20 PROM1
1	140205123	1.89E-03	Nonsynonymous exon 20 ATP8B4
1	139685697	2.82E-03	Nonsynonymous exon 32 TRPM7
14	16830511	5.34E-03	Intron HMMR
14	16840478	8.05E-03	Intron NUDCD2
4	5924012	9.70E-03	Nonsynonymous exon 4 ATXN7L1
21	48664783	1.13E-02	Intron CTNND2
10	57209370	1.83E-02	Downstream PDSS2
5	77536297	1.89E-02	Nonsynonymous exon 8 CLCA2
5	78709303	2.27E-02	Nonsynonymous exon 4 WDR63
1	139944477	4.16E-02	Nonsynonymous exon 10 SLC27A2
10	56817838	4.47E-02	Intron QRSL1
14	37281732	4.93E-02	Downstrream SPATA24

*Chr* chromosome, *bp* base pair

## Discussion

GWA analysis in our discovery cohort of U.S. Standardbreds identified 12 SNP markers within two different loci on ECA14 that were moderately associated (*p* ≤ 1.86 × 10^−4^) with OC status. These regions have not been identified as significantly associated with OC in any previously published GWAS (the previously reported association on ECA14 in French Trotters spanned a region from 67 to 79 Mb [37]). This population differed from those in previous OC GWAS in that there was a shared early environment between cases and controls, thus reducing the confounding effects of management factors such as diet and exercise. An additional advantage of this cohort was that individuals were closely related, thus potentially enhancing the number of risk alleles within the population and improving the power of the GWA to detect association with disease [38, 39]. However, the sample size was still relatively limited, which may be why genome-wide significance was not achieved. The use of mixed model analysis in GEMMA allowed both for correction for population structure [40] using a marker-based relatedness matrix [41] and inclusion of covariates that may play a role in disease development, such as gait [42].

SNPs are chosen for inclusion in genotyping panels based on their distribution across the genome and their frequency within the population rather than on their locations within protein-coding genes. Of the five most significantly associated SNPs in the GWAS reported here (chr14.16401778, chr14.34284113, chr14.17858976, chr14.17866794, chr14.17626659), three are in very large introns and two are intergenic, so these SNPs are likely “tagging” true risk variants with which they are in linkage disequilibrium (LD) [43, 44]. Horses exhibit extensive LD, and Standardbreds in particular have the greatest long range LD (>1,200 kb) among horse breeds [45]. Thus, it is reasonable that a SNP demonstrated to be associated with disease in our GWAS could be reflecting the effects of a risk variant up to 1 Mb distant (or farther) from that SNP marker. Due to the large number of potential candidate genes within 1 Mb of the regions of interest, and the extensive LD in Standardbreds, we chose to perform whole-genome sequencing in a subset of our discovery cohort for variant discovery. One advantage of this approach, beyond its efficiency in cataloging both coding and non-coding variants, was that it allowed variant discovery to be carried out in eighteen horses, rather than just two or three. This resulted in a more complete picture of what variants were present in the population, as well as a better estimate of how these variants segregate with disease status, which helped with prioritization for follow-up in the larger group.

We investigated variants both within regions of the genome corresponding to GWAS findings in our discovery cohort as well as from selected additional regions published by others as putative quantitative trait loci (QTLs) for hock OC. Genomic heritability calculations indicate that together these variants explain 10–20 % of the phenotypic variance for this trait. Overall, SNPs from four loci on three chromosomes (ECA14, 10 and 21) were associated with disease status in both the discovery and validation cohorts. The association of putative risk alleles from within the same regions with disease status in two independently sampled populations of Standardbreds suggest that these are true risk loci in this breed. Population-specific risk factors may still exist (i.e. variants on ECA1, 3 and 5 in the validation population) and will need to be investigated in future studies.

Additional investigation of variants within the risk loci on ECA14, 10 and 21 will need to be carried out in the populations reported here to identify specific risk alleles. There are several genes within the identified risk loci that are plausible biologic candidates for playing a role in OC and which contained variants that were highly associated with disease in either the discovery or the validation cohort, including *MAT2B*, *HMMR*, *CCNG1* and *ARHGAP26*. Methionine adenosyltransferase II beta (*MAT2B*) catalyzes the synthesis of S-adenosylmethionine (SAMe). Methionine is an essential amino acid in normal skeletogenesis [46], and exogenous SAMe is utilized therapeutically for osteoarthritis because of its beneficial effects on cartilage, including increased proteoglycan synthesis [47]. Hyaluronan-mediated motility receptor (*HMMR*) is a hyaluronan-binding protein that has been identified in epiphyseal cartilage, articular cartilage and interzone cells (located in what will become the joint space) in the developing joints of embryonic chicks, and is believed to play a major role in synovial joint formation [48]. The role of cyclin G1 (*CCNG1*) in cartilage has not been reported, but members of the cyclin family have been reported to regulate chondrocyte proliferation [49, 50], and cyclin-dependent kinase inhibitors have been shown to mediate growth arrest in chondrocytes [51]. GTPase activating proteins, such as rho GTPase activating protein 26 (*ARHGAP26*), are crucial mediators of the activity of Rho GTPases, which play an important role in chondrocyte differentiation and normal long bone development [52]. Clearly, variants in these genes will be of particular interest. However, in addition to variants within or near protein-coding genes, which have been our focus to date, we may need to consider a possible role for variants within non-coding/regulatory regions of the genome as we move forward.

## Conclusions

Here we report the discovery and validation of risk loci on ECA14 for tarsal OC in Standardbred horses. Additional putative risk loci were identified on ECA10 and 21, although these were less significantly associated with disease status. Together, the investigated variants explain 10–20 % of the phenotypic variance of OC in the reported population. This is the first report of a GWA analysis in a cohort of horses specifically selected for a shared early environment, the first to use imputation to greatly increase the number of available genotypes in the GWA population, and the first report validating putative risk loci for equine OC in an independent population. Further evaluation of these risk loci should be attempted in Standardbreds with OC lesions in joints other than the tarsus, and in another breed (i.e. Warmblood) affected by tarsal OC. This would help to determine if the identified risk loci are specific to the Standardbred breed, or to tarsal OC or are universal risk loci for OC (i.e. across all predilection sites and breeds).

Additional investigation is needed to identify the actual functional variants underlying disease risk within the validated risk loci. This is the first step towards the long-term goal of constructing a genetic risk model for OC that allows for genetic testing and quantification of risk in individual horses. This risk model could contain as many as 6–15 putative risk alleles, similar to those that have been used successfully to predict recurrence and survival in patients with breast cancer, another genetically complex disorder [53]. Improved risk assessment will facilitate management changes and early intervention in high-risk horses and allow for informed breeding decisions in high-risk pedigrees that will ultimately help to reduce disease prevalence.

## Methods

### GWAS discovery cohort

This study was conducted under the approval of the University of Minnesota Institutional Animal Care and Use Committee (protocol #1111B07139). The discovery cohort was comprised of 182 Standardbred yearlings born and raised on a single breeding farm in the eastern United States. Individuals were included from the 2007 (*n* = 94), 2009 (*n* = 16), 2010 (*n* = 52), and 2012 (*n* = 20) foal crops. Management practices, including diet and exercise regimen, were the same for all foals at this facility during their first year of life. Average prevalence of tarsal OC on this farm for the included foal crops was 28 %. Yearlings were identified for inclusion in this study during preparation for one of several breed-recognized sales events. Affected horses (*n* = 70) had surgically-confirmed OC lesions of one or both tarsi. Of the 112 age-matched related controls, 78 were radiographically confirmed to be free of tarsal OC, while 34 (from the 2007 foal crop) were presumed unaffected because of lack of clinical signs including effusion and lameness.

### DNA isolation and whole-genome genotyping

DNA was isolated from blood (2007 and 2012 foals) or hair roots (2009 and 2010 foals) samples using the Gentra® Puregene® Blood Kit (Qiagen, Valencia, CA) per manufacturer recommendations. Briefly, for blood samples, RBC lysis solution was added to samples at a 3:1 ratio, incubated and centrifuged. After discarding the supernatant, cell lysis solution was added to the white blood cell pellet and the cells were re-suspended, after which protein was precipitated and discarded. DNA was precipitated in isopropanol and subsequently washed in ethanol prior to final hydration. A similar protocol was followed for hair root samples, omitting the RBC lysis step. Quantity and purity of extracted DNA were assessed using spectrophotometric readings at 260 and 280 nm (NanoDrop 1000, Thermo Scientific, Wilmington, DE).

Genome-wide genotyping of single nucleotide polymorphism (SNP) markers was performed by Neogen GeneSeek (Lincoln, NE) using the Illumina Custom Infinium SNP genotyping platform. Samples from the 2007 foal crop were genotyped at 54,602 SNPs using the first generation Illumina Equine SNP50 chip, while the remaining samples were genotyped at 65,157 SNP markers using the second generation Illumina Equine SNP70 chip.

### Genotype imputation

The two equine genotyping platforms used in the discovery population share 45,703 SNPs. This shared set of markers can be extracted and the files merged into a single data set, but data from tens of thousands of markers is lost. Genotype imputation is a technique that statistically estimates genotypes from non-assayed SNPs by comparing haplotype blocks in the study population with haplotype blocks in a more densely genotyped reference population. A pipeline for imputation of equine genotyping data was established and validated utilizing BEAGLE (version 3) [54] software for imputation [33]. Using this pipeline, imputation was performed in the 2007 cohort for the ~18,000 markers unique to the Equine SNP70 chip, while imputation was performed in the remaining samples for the ~9,000 markers unique to the Equine SNP50 chip. Resulting imputed files were merged with the original data files using the --merge command in PLINK [55].

A new custom equine genotyping platform containing ~670,000 SNPs (670 k; Affymetrix Axiom Array) became available commercially in January 2015. These SNPs were selected from an initial list of ~2 million SNPs identified by whole-genome sequencing in 166 horses from 32 diverse breeds [56]. Imputation using BEAGLE (version 4.0) as described above was performed for the entire discovery cohort, using a mixed breed reference population (*n* = 513) in a stepwise fashion. First, the original SNP50/SNP70 data was imputed to the SNPs included in the new 670 k array. SNPs from this step were then pruned to those correctly called > 95 % of the time in horses genotyped on both platforms (SNP50/SNP70 and 670 k; *n* = 40) and subsequently imputed to 2 million SNPs. These SNPs were again pruned at > 95 % concordance for use in analyses.

### Genome-Wide Association (GWA) analysis

Initial GWA analysis was carried out after imputation (between the SNP50 and SNP70 chips) using GEMMA (Genome-wide Efficient Mixed Model Analysis) software [57]. The GWA was performed using the options to create a centered relatedness matrix (−gk 2) and perform all three possible frequentist tests: Wald, likelihood ratio and score (−fa 4). A covariate file (−c) including sex and gait (pacer or trotter) was incorporated into the mixed model. The relatedness matrix was incorporated to control for family structure among the discovery cohort and was constructed using a linkage-disequilibrium (LD)-pruned set of markers from the imputed genome-wide SNP data (100 SNP windows, sliding by 25 SNPs along the genome, pruned at r^2^ > 0.2; PLINK command --indep-pairwise 100 25 0.2) [45]. SNPs were pruned prior to GWA using the default GEMMA parameters of MAF < 1 % and missingness < 95 %. The genome-wide significance cutoff using an adjusted Bonferroni correction based on the effective number of independent tests in our data was *p* <1.86 × 10^−6^ [58]. *P*-values between 1.68 × 10^−4^ and 1.68 × 10^−6^ were considered to indicate moderate association.

Haplotype analysis was conducted on 61,046 marker set (used in the GEMMA analysis described above) using Haploview [59]. Haplotype blocks were constructed (− blockoutput) using the default algorithm taken from Gabriel et al. [60], which is based on 95 % confidence bounds on D prime. Case control association testing (− assocCC) was performed with 1000 permutations (− permtests 1000). Permuted associations were considered statistically significant at *p* < 0.05.

### Whole-genome sequencing

Individuals were selected for whole-genome sequencing on the basis of haplotype analysis within regions of interest on equine (ECA) chromosomes 2, 6 and 14. Haplotypes were evaluated for both their absolute frequency within the OC-affected and OC-unaffected groups and for differences in frequency between groups. For each region, the most common haplotype within an affectation status that also exhibited a large difference between OC-affected and OC-unaffected groups was selected as the haplotype of interest. Individuals that exhibited these haplotypes in one or more of the regions of interest were eligible for selection for whole-genome sequencing. Horses were preferentially selected if they had the haplotype of interest in more than one region of interest; however, consideration was also given to balancing the selected cohort by sex, gait (pace or trot) and sire to manage potential confounding factors.

Genomic DNA (2–6 μg) from the 18 selected horses was submitted to the University of Minnesota Biomedical Genomics Center (UMGC) for quality control, library preparation and sequencing. Samples were subjected to library preparation including fragmentation, polishing, and adaptor ligation and were prepared with an indexed barcode for a 100 bp paired-end run on the Illumina HiSeq sequencer, per standard protocols. Of the nine affected horses, 3 were sequenced at 12x coverage and 6 at 6x coverage; the same distribution was used for the nine unaffected horses.

Data analysis, including quality control, alignment and variant detection, was carried out following published best practices [61] within the Galaxy framework hosted by the Minnesota Supercomputing Institute. Briefly, reads that passed quality control were mapped to the reference sequence (EquCab 2.0, Sept. 2007) using BWA for Illumina. Ambiguously mapped reads, low quality reads and PCR duplicates were removed, after which reads were realigned around indels. Base quality recalibration was performed to remove systematic bias. This process was completed for the reads from each of the eight lanes for every individual before merging the mapped and recalibrated “lane-level” BAM files into a single “sample-level” file. Removal of duplicates and realignment around indels was repeated on the merged file. The eighteen sample-level files were merged into three groups of six, evenly divided between affected and unaffected individuals, for the purposes of variant calling using the UnifiedGenotyper utility of the Broad Institute’s Genome Analysis ToolKit (GATK) with a threshold phred-scale score of 20.0. Variants were filtered using the following thresholds: Quality Depth (QD) < 2.0 (assesses variant quality score taking into account depth of coverage at that variant), Read Position Rank Sum < −20.0 (Mann-Whitney Rank Sum test on the distance of the variant from the end of each read covering it), Fisher Strand (FS) > 200.0 (phred-scaled *p*-value to detect strand bias). Filtered variant lists from the three groups were combined into a single variant calling file (VCF) for subsequent analysis. Predicted functional effect for each called variant was determined based on the current equine reference genome annotation using SnpEff [62]. Frequency of variants within cases and controls, and the significance of frequency differences, was calculated using SnpSift CaseControl [63]. Variants from particular chromosomal regions of interest were selected using SnpSift Intervals and converted into Excel format for further evaluation.

### Sequenom assay

A custom Sequenom assay was designed for high-throughput genotyping of prioritized variants. Variants were selected from within the top chromosomal regions of interest from the GWA in the discovery cohort as well as from chromosomal regions previously reported to be associated with hock OC (see Background). Variants discovered through whole-genome sequencing were filtered to include SNPs that passed all quality control filters, and were subsequently prioritized according to the following parameters: 1) present in 3+ more cases than controls, or vice versa; 2) not intergenic; 3) non-synonymous, then synonymous changes; 4) if intronic, close to intron-exon boundary (preferably <100 bp); 5) coding genes preferred over non-coding; and 6) if upstream/downstream, as close as possible to start/stop codon.

An attempt was made to include at least one variant per coding gene within each region of interest; if multiple variants of equally low predicted function were the only ones available within a gene, then the one with the higher genomic *p*-value was selected. In addition to the experimental SNPs, 98 ancestry informative markers (AIMs) were included in the Sequenom assay to help control for population structure [34]. The AIMs used in this study were generated from a large population of gaited horses, including Standardbreds and capture 97.6 % of the variation captured by significant principal components from genome-wide genotyping data.

Genotyping results were analyzed using the GEMMA parameters described previously (see *Genome-Wide Association (GWA) Analysis*). The relatedness matrix was constructed using the AIMs.

To determine the relative amount of phenotypic variation explained by prioritized variants, genomic heritability was etimated using a genomic best linear unbiased predictor (GBLUP) analysis (SNP & Variation Suite 8, Golden Helix, Bozeman, MT). Assuming an additive genetic model, GBLUP uses a genomic relationship matrix (GRM) derived from genotype data to predicts allele substitution effects of each marker and additive genetic effect of each individual [64]. Covariates of sex and gait were included in the GBLUP analysis.

### Validation cohort

The horses included in the validation cohort have been described in detail by Lykkjen et al. [36] This population included 162 Norwegian Standardbred trotter yearlings belonging to 22 half-sibling families that were identified as either affected or unaffected with tarsal OC based on survey radiographs taken between 8 and 18 months of age (mean 12.1mo). Horses with simultaneous lesions in the fetlock were removed so that the OC phenotype would be comparable to the discovery population, resulting in a final validation cohort of 139 horses (60 cases, 79 controls). These horses were genotyped on the custom Sequenom assay (see *Sequenom assay*), which included SNPs from chromosomal regions of interest previously reported in a GWA in this same population (ECA1 and 3). Genotyping results were analyzed using the GEMMA parameters described previously (see *Genome-Wide Association (GWA) Analysis*). The relatedness matrix was constructed using the AIMs.

## Availability of supporting data

The data sets supporting the results of this article, including whole-genome SNP data in the discovery cohort and Sequenom genotyping data in both the discovery and validation cohorts are available in the NRSP-8 Bioinformatics Data Repository, URL: http://www.animalgenome.org/repository/pub/IL2015.0629/. Whole-genome sequencing data is available in the NCBI Sequence Read Archive (SRA) SRP067684 (BioProject PRJNA306677).

## Additional file

Additional file 1: Table S1. Named genes located within the top regions of association on ECA14 from the GWA analysis. **Table S2.** Haplotype analysis within the top regions of association on ECA14 from the GWA analysis. **Table S3.** Top GWA SNPs from GEMMA mixed model analysis of data imputed to 670 k and 2 M SNP lists. **Table S4.** Summary of variants by type and region. **Table S5.** Regions of interest for which detailed annotation of SNPs was performed. **Table S6.** Putative risk variants for OC that were selected for inclusion in the custom Sequenom genotyping assay (*n* = 240). **Table S7.** Frequency of alternate allele in cases and controls for each SNP in the Sequenom platform that genotyped successfully in the discovery or validation populations. (DOCX 93 kb)

## Abbreviations

AIM: Ancestry informative marker
DNA: Deoxyribonucleic acid
ECA: *Equus caballus* (equine)
GABA: Gamma-aminobutyric acid
GATK: Genome analysis toolkit
GBLUP: Genomic best linear unbiased predictor
GEMMA: Genome-wide efficient mixed model analysis
GRM: Genomic relationship matrix
GWA(S): Genome-wide association (study)
LD: Linkage disequilibrium
MAF: Minor allele frequency
OC(D): Osteochondrosis (dissecans)
QTL: Quantitative trait locus
RBC: Red blood cell
SAMe: S-adenosylmethionine
SNP: Single nucleotide polymporphism
UMGC: University of Minnesota Biomedical Genomics Center
VCF: Variant calling file
