# Supplementary material for: Identification and validation of risk loci for osteochondrosis in standardbreds
Source: BMC Genomics. 2016 Jan 12;17:41. doi: 10.1186/s12864-016-2385-z (PMC4709891; doi:10.1186/s12864-016-2385-z)
Supplement: Additional file 1: Table S1. — Named genes located within the top regions of association on ECA14 from the GWA analysis. Table S2. Haplotype analysis within the top regions of association on ECA14 from the GWA analysis. Table S3. Top GWA SNPs from GEMMA mixed model analysis of data imputed to 670 k and 2 M SNP lists. Table S4. Summary of variants by type and region. Table S5. Regions of interest for which detailed annotation of SNPs was performed. Table S6. Putative risk variants for OC that were selected for inclusion in the custom Sequenom genotyping assay (n = 240). Table S7. Frequency of alternate allele in cases and controls for each SNP in the Sequenom platform that genotyped successfully in the discovery or validation populations. (DOCX 93 kb) [file 12864_2016_2385_MOESM1_ESM.docx]

**Table S1:** Named genes located within the top regions of association on ECA14 from the GWA analysis. Markers in **bold** are moderately associated with OC, with p-values ≤ 1.68 x 10^-4^ (**Table 1**). Genes in **bold** are considered potential candidate genes based on annotated function. Only predicted protein-coding genes are listed.

| Region on ECA14 | Markers | Genes within region | Genes within 1Mb of region |
| --- | --- | --- | --- |
| ~16.4-18.3Mb | **14.16401778** | ***MAT2B****,* ***HMMR****, NUDCD2,* ***CCNG1****, GABRG2, GABRA1, GABRA6, GABRB2* | *ATP10B* |
|  | **14.17534553** |  |  |
|  | **14.17626659** |  |  |
|  | **14.17858976** |  |  |
|  | **14.17866794** |  |  |
|  | 14.18305845 |  |  |
| ~33.6-36.2Mb | **14.33630011** | ***NR3C1****,* ***ARHGAP26****,* ***FGF1****, SPRY4, GNPDA1, NDFIP1, KIAA0141, RNF14, PCDH12, PCDH1, ARAP3, FCHSD1, RELL2,* ***HDAC3****, DIAPH1, PCDHGC5, PCDHGB4, PCDHGA2, PCDHGA1, SLC25A2, PCDHB10, PCDHB16, PCDHB7, PCDHB2, PCDHA12, PCDHB14, PCDHB15, PCDHB3, PCDHB1, PCDHA3, PCDHA1, PCDHAC2, PCDHB11, PCDHA8* | *NDUFA2, SRA1, ZMAT2, CD14, ANKHD1, HARS2, HARS, DND1, WDR55, TMCO6,* ***SLC35A4****, APBB3, SLC4A9, HBEGF, CYSTM1, PFDN1, NRG2, PSD2, CXXC5, UBE2D2, KCTD16, YIPF5* |
|  | **14.34284113** |  |  |
|  | **14.34366588** |  |  |
|  | **14.36214363** |  |  |

**Table S2:** Haplotype analysis within the top regions of association on ECA14 from the GWA analysis. Case-control association testing was performed with 1000 permutations, with significance set at p < 0.05 (haplotypes in **bold**).

| Region on ECA14 | Haplotype Block | Markers | Haplotypes | Haplotype Frequency | Case Frequency | Control Frequency | p-value | permuted p-value |
| --- | --- | --- | --- | --- | --- | --- | --- | --- |
| ~16.4-18.3Mb | 1 | chr14.16656064  chr14.16671923 | AC  GC  AA | 0.428  0.398  0.174 | 0.355  0.493  0.152 | 0.473  0.339  0.188 | 0.0274  0.0038  0.3892 | 0.47  0.08  1 |
|  | 2 | chr14.16812672  chr14.16836406  chr14.16839269 | ACG  GAA  AAA | 0.829  0.146  0.014 | 0.79  0.188  0.022 | 0.853  0.12  0.009 | 0.1233  0.0742  0.3296 | 0.9  0.753  1 |
|  | 3 | chr14.16871893  chr14.16871997  chr14.16872114  chr14.16883335  chr14.16948854  chr14.17003700 | AGAGGA  GAGGGA  GAGAAC  GAGAGA  GAGGGC | 0.665  0.169  0.127  0.017  0.016 | 0.622  0.168  0.174  0.014  0.02 | 0.692  0.17  0.098  0.018  0.013 | 0.1724  0.9596  0.0357  0.8076  0.5823 | 0.967  1  0.537  1  1 |
|  | 4 | chr14.17031993  chr14.17035906  chr14.17120130  chr14.17157018  chr14.17222679  chr14.17223210  chr14.17262735 | AGAAC  AGGGC  GGGGC  GAGGC  GAGGA | 0.448  0.312  0.11  0.075  0.055 | 0.384  0.355  0.087  0.101  0.072 | 0.487  0.286  0.125  0.058  0.045 | 0.0567  0.1666  0.2621  0.1268  0.2605 | 0.678  0.957  0.998  0.909  0.998 |
|  | 5 | chr14.17586672  chr14.17590353  chr14.17590481  chr14.17626659  chr14.17636198  chr14.17640793  chr14.17713486  chr14.17756907  chr14.17758781 | CGCAAAGG  **CGCGAAAA**  AAAAGGAA  CGCGAGAA  CGCAAGAA  CGCAAAAA | 0.566  **0.152**  0.113  0.075  0.074  0.02 | 0.623  **0.072**  0.138  0.052  0.101  0.015 | 0.531  **0.201**  0.098  0.089  0.058  0.022 | 0.0865  **9.0E-4**  0.2498  0.1854  0.1335  0.6417 | 0.825  **0.021**  0.997  0.979  0.919  1 |
|  | 6 | chr14.17858976  chr14.17866162  chr14.17866282 | CGA  CAG  AGA  CGG | 0.533  0.282  **0.141**  0.044 | 0.58  0.297  **0.065**  0.058 | 0.504  0.272  **0.188**  0.036 | 0.1634  0.6107  **0.0012**  0.317 | 0.954  1  **0.029**  1 |
|  | 7 | chr14.17866794  chr14.17934824  chr14.17941548  chr14.18057043  chr14.18066309  chr14.18077886 | GAAAAA  GAAGAA  GAAGAG  GGGGGA  **AAAGAG** | 0.34  0.201  0.161  0.155  **0.134** | 0.37  0.217  0.175  0.174  **0.064** | 0.321  0.191  0.153  0.143  **0.178** | 0.3476  0.5546  0.5813  0.4274  **0.0022** | 1  1  1  1  **0.047** |
|  | 8 | chr14.18257709  chr14.18257717 | GG  AA | 0.801  0.199 | 0.804  0.196 | 0.799  0.201 | 0.9034  0.9034 | 1  1 |
|  | 9 | chr14.18305845  chr14.18305965 | CG  CA  AG | 0.445  0.343  0.213 | 0.464  0.391  0.145 | 0.433  0.312  0.254 | 0.5677  0.1249  0.0134 | 1  0.901  0.273 |
| ~33.6-36.2Mb | 1 | chr14.33026614  chr14.33037541 | GG  AA | 0.724  0.276 | 0.688  0.312 | 0.746  0.254 | 0.2377  0.2377 | 1  1 |
|  | 2 | chr14.33127735  chr14.33129361 | AA  GG | 0.950  0.050 | 0.971  0.029 | 0.938  0.062 | 0.1542  0.1542 | 0.979  0.979 |
|  | 3 | chr14.33196047  chr14.33209248  chr14.33345601  chr14.33348259  chr14.33369846  chr14.33438125  chr14.33515533 | CGGACAA  AAGACGA  CGAGAAG  AAGACAA  CGGACAG  CGGACGA | 0.461  0.236  0.152  0.052  0.050  0.021 | 0.466  0.223  0.138  0.077  0.029  0.023 | 0.458  0.244  0.161  0.037  0.063  0.019 | 0.8811  0.6498  0.5532  0.0958  0.1515  0.8023 | 1  1  1  0.893  0.977  1 |
|  | 4 | chr14.33522408  chr14.33534828  chr14.33539294 | AAG  AGA  GAG | 0.401  0.334  0.265 | 0.355  0.391  0.254 | 0.429  0.299  0.272 | 0.1657  0.07090.6955 | 0.982  0.835  1 |
|  | 5 | chr14.33718981  chr14.33767090  chr14.33820686  chr14.33868321 | GAAG  GCGA  AAAG  GCAA | 0.409  0.246  0.235  0.108 | 0.464  0.210  0.217  0.101 | 0.375  0.268  0.246  0.112 | 0.0952  0.2155  0.5395  0.7621 | 0.893  0.998  1  1 |
|  | 6 | chr14.34195019  chr14.34280553  chr14.34284113  chr14.34366588  chr14.34371665  chr14.34425422  chr14.34490992  chr14.34500330  chr14.34526616  chr14.34617652 | GAACGGAGGG  AGACAAAGGG  GAACAGGAGA  GAGAGGAGAG  GAGAGGAGGG | 0.309  0.304  0.232  0.069  0.060 | 0.268  0.297  0.196  0.116  0.094 | 0.335  0.308  0.254  0.040  0.040 | 0.1824  0.8261  0.1979  0.0058  0.0343 | 0.987  1  0.988  0.129  0.534 |
|  | 7 | chr14.34629185  chr14.34638831  chr14.34639487  chr14.34654537 | GGAG  AAGG  AAGA  GAGG | 0.564  0.345  0.069  0.022 | 0.558  0.304  0.109  0.029 | 0.567  0.371  0.045  0.018 | 0.8669  0.1983  0.0196  0.4842 | 1  0.988  0.371  1 |
|  | 8 | chr14.34827154  chr14.34874031  chr14.34936564  chr14.34939265  chr14.34939671  chr14.34939810  chr14.34967660 | AGAGCAG  GGAGCAG  GGGAAAA  GGGAAGA  GAGGCAG  GGGGCAG | 0.351  0.232  0.171  0.138  0.074  0.034 | 0.304  0.225  0.203  0.109  0.116  0.043 | 0.379  0.237  0.152  0.156  0.048  0.028 | 0.1458  0.7933  0.21  0.2028  0.0158  0.436 | 0.973  1  0.998  0.993  0.319  1 |
|  | 9 | chr14.35091406  chr14.35097879  chr14.35101498  chr14.35101570  chr14.35103675 | GAAGA  GAGAG  AAGAG  GGAGA | 0.745  0.123  0.072  0.059 | 0.684  0.099  0.116  0.099 | 0.782  0.138  0.045  0.035 | 0.0382  0.27  0.0107  0.013 | 0.591  1  0.257  0.284 |
|  | 10 | chr14.35112726  chr14.35162634  chr14.35162977  chr14.35176242  chr14.35202529  chr14.35223848 | AGGAGG  GGGGGA  AGGGGA  AAAAAA  AAGAGA | 0.533  0.304  0.072  0.058  0.022 | 0.486  0.261  0.116  0.101  0.029 | 0.562  0.330  0.045  0.031  0.018 | 0.1538  0.1627  0.0107  0.0055  0.4842 | 0.978  0.981  0.257  0.12  1 |
|  | 11 | chr14.35305157  chr14.35361117  chr14.35365631  chr14.35383705  chr14.35491369  chr14.35508045  chr14.35519776  chr14.35556745  chr14.35603146  chr14.35614844 | GACGGAAAAA  AGCAGAGAGG  AACAGAGAGG  AAAAGGGCGG  AACAAAGAGG | 0.384  0.216  0.198  0.097  0.086 | 0.377  0.257  0.158  0.137  0.062 | 0.388  0.190  0.223  0.071  0.101 | 0.8235  0.1289  0.137  0.0388  0.2043 | 1  0.962  0.967  0.591  0.993 |
|  | 12 | chr14.35669710  chr14.35669868 | CA  AG | 0.506  0.494 | 0.471  0.529 | 0.527  0.473 | 0.3026  0.3026 | 1  1 |
|  | 13 | chr14.35671602  chr14.35672405  chr14.35678329  chr14.35687375 | AAAC  CGCA  CAAC  CGCC  CGAC | 0.395  0.390  0.077  0.077  0.061 | 0.377  0.326  0.109  0.123  0.065 | 0.406  0.429  0.058  0.049  0.058 | 0.5779  0.0521  0.0797  0.0104  0.7812 | 1  0.685  0.863  0.233  1 |
|  | 14 | chr14.35928173  chr14.35944378  chr14.35953786 | AAG  GGA  GAA | 0.395  0.320  0.285 | 0.377  0.399  0.225 | 0.406  0.272  0.321 | 0.5779  0.01240.0474 | 1  0.272  0.653 |
|  | 15 | chr14.36147105  chr14.36151779 | AA  GG | 0.699  0.301 | 0.739  0.261 | 0.674  0.326 | 0.1903  0.1903 | 0.988  0.988 |
|  | 16 | chr14.36214363  chr14.36243733  chr14.36243867  chr14.36308621  chr14.36309843  chr14.36414548  chr14.36415048  chr14.36460213 | GGGGAGAG  GGAGGGGG  GGAGGAGA  AAAAGAGA  AGAGGAGA  GGAGGAGG | 0.401  0.381  0.108  0.077  0.019  0.011 | 0.384  0.312  0.123  0.123  0.036  0.014 | 0.411  0.424  0.098  0.049  0.009  0.009 | 0.6152  0.0323  0.4567  0.0104  0.0669  0.6228 | 1  0.519  1  0.233  0.777  1 |
|  | 17 | chr14.36484340  chr14.36525721  chr14.36580936  chr14.36588085 | CGAG  AGGA  **AAGA**  CGGA  CGAA | 0.395  0.301  **0.149**  0.120  0.036 | 0.376  0.261  **0.225**  0.098  0.041 | 0.406  0.326  **0.103**  0.133  0.032 | 0.5625  0.1903  **0.0016**  0.3221  0.6722 | 1  0.988  **0.033**  1  1 |
|  | 18 | chr14.36766817  chr14.36771713 | GG  GA  AA | 0.403  0.376  0.221 | 0.384  0.420  0.196 | 0.415  0.348  0.237 | 0.5577  0.1691  0.3617 | 1  0.982  1 |
|  | 19 | chr14.37021304  chr14.37040197  chr14.37047668  chr14.37078210  chr14.37079127  chr14.37086220  chr14.37142927  chr14.37150472 | CGAAGGG  CGAGAGA  AAAAGGA  CGGGAAA | 0.491  0.290  0.134  0.072 | 0.442  0.290  0.138  0.116 | 0.521  0.290  0.131  0.045 | 0.1466  0.9941  0.8599  0.0107 | 0.974  1  1  0.257 |
|  | 20 | chr14.37323984  chr14.37339549 | AA  GG | 0.878  0.122 | 0.877  0.123 | 0.879  0.121 | 0.9402  0.9402 | 1  1 |
|  | 21 | chr14.37431608  chr14.37464197  chr14.37482906  chr14.37597522  chr14.37649533  chr14.37682820  chr14.37703245  chr14.37762830  chr14.37800197  chr14.37845062  chr14.37867562  chr14.37915758 | ACAGAAGAACGG  ACAAAAGAGCGG  GAGGGAGGAAGG  GAGGGCGGACGG  GAGGGAGAACAA  ACAAAAAAACGG  ACAAAAGAACGG | 0.237  0.219  0.171  0.124  0.124  0.072  0.032 | 0.224  0.223  0.130  0.138  0.123  0.114  0.033 | 0.245  0.217  0.196  0.116  0.125  0.046  0.032 | 0.6495  0.9028  0.1055  0.545  0.9595  0.0147  0.9418 | 1  1  0.903  1  1  1  0.304 |
|  | 22 | chr14.37994170  chr14.38006306 | AA  GG | 0.820  0.180 | 0.862  0.138 | 0.795  0.205 | 0.1032  0.1032 | 0.9  0.9 |

**Table S3:** Top GWA SNPs from GEMMA mixed model analysis of data imputed to 670k and 2M SNP lists. Uncorrected p-values are presented for the Wald test, the Likelihood ratio test (lrt) and the Score test. SNPs in **bold** for each analysis are moderately associated with disease (p-values ≤ 5 x 10^-5^ as calculated by the likelihood ratio test). SNPs with a p-value < 5 x 10^-4^ in both analyses are highlighted. Chr = chromosome; bp = base pair.

| **670k set**  **(123,352 markers available for analysis)** | | | | |  | **2M set**  **(243,115 markers available for analysis)** | | | | |
| --- | --- | --- | --- | --- | --- | --- | --- | --- | --- | --- |
| **Chr** | **bp** | **P-value**  **wald** | **P-value**  **lrt** | **P-value**  **score** |  | **Chr** | **bp** | **P-value**  **wald** | **P-value**  **lrt** | **P-value**  **score** |
| **14** | **16388236** | 5.54E-06 | 4.22E-06 | 1.39E-05 |  | **14** | **16419200** | 4.07E-06 | 3.08E-06 | 1.08E-05 |
| **14** | **16401778** | 5.54E-06 | 4.22E-06 | 1.39E-05 |  | **14** | **16351763** | 5.54E-06 | 4.22E-06 | 1.39E-05 |
| **14** | **16419200** | 1.05E-05 | 8.10E-06 | 2.35E-05 |  | **14** | **16388236** | 5.54E-06 | 4.22E-06 | 1.39E-05 |
| **14** | **17794493** | 1.58E-05 | 1.23E-05 | 3.31E-05 |  | **14** | **16401778** | 5.54E-06 | 4.22E-06 | 1.39E-05 |
| **14** | **17858976** | 1.89E-05 | 1.49E-05 | 3.86E-05 |  | **14** | **16418359** | 5.54E-06 | 4.22E-06 | 1.39E-05 |
| **14** | **17866794** | 1.89E-05 | 1.49E-05 | 3.86E-05 |  | **14** | **16436852** | 5.54E-06 | 4.22E-06 | 1.39E-05 |
| **14** | **17896951** | 1.89E-05 | 1.49E-05 | 3.86E-05 |  | **14** | **16398821** | 6.27E-06 | 4.84E-06 | 1.55E-05 |
| **14** | **17599786** | 3.50E-05 | 2.79E-05 | 6.50E-05 |  | **14** | **17305612** | 7.12E-06 | 5.45E-06 | 1.71E-05 |
| **14** | **17534553** | 4.11E-05 | 3.28E-05 | 7.45E-05 |  | **14** | **17313751** | 7.12E-06 | 5.45E-06 | 1.71E-05 |
| **14** | **17626659** | 4.11E-05 | 3.28E-05 | 7.45E-05 |  | **14** | **17379670** | 8.39E-06 | 6.45E-06 | 1.96E-05 |
| 14 | 34366588 | 7.56E-05 | 6.13E-05 | 1.26E-04 |  | **14** | **17403034** | 8.39E-06 | 6.45E-06 | 1.96E-05 |
| 14 | 17380047 | 1.02E-04 | 8.48E-05 | 1.66E-04 |  | **14** | **17420872** | 8.39E-06 | 6.45E-06 | 1.96E-05 |
| 14 | 33630011 | 1.24E-04 | 1.02E-04 | 1.93E-04 |  | **14** | **17763440** | 8.69E-06 | 6.69E-06 | 2.02E-05 |
| 14 | 36214363 | 2.07E-04 | 1.72E-04 | 3.04E-04 |  | **14** | **17794493** | 1.58E-05 | 1.23E-05 | 3.31E-05 |
| 4 | 28769871 | 2.39E-04 | 1.99E-04 | 3.44E-04 |  | **14** | **17808170** | 1.58E-05 | 1.23E-05 | 3.31E-05 |
| 21 | 48322513 | 2.46E-04 | 2.05E-04 | 3.53E-04 |  | **14** | **17876909** | 1.58E-05 | 1.23E-05 | 3.31E-05 |
| 23 | 40127611 | 2.52E-04 | 2.11E-04 | 3.61E-04 |  | **14** | **17877973** | 1.58E-05 | 1.23E-05 | 3.31E-05 |
| 15 | 28129429 | 2.50E-04 | 2.44E-04 | 4.10E-04 |  | **14** | **17848042** | 1.89E-05 | 1.49E-05 | 3.86E-05 |
| 1 | 118288481 | 2.81E-04 | 2.44E-04 | 4.11E-04 |  | **14** | **17858976** | 1.89E-05 | 1.49E-05 | 3.86E-05 |
| 15 | 28682409 | 2.76E-04 | 2.53E-04 | 4.23E-04 |  | **14** | **17866794** | 1.89E-05 | 1.49E-05 | 3.86E-05 |
| 14 | 34127696 | 3.07E-04 | 2.57E-04 | 4.29E-04 |  | **14** | **17482785** | 2.45E-05 | 1.94E-05 | 4.80E-05 |
| 21 | 47435380 | 3.19E-04 | 2.78E-04 | 4.60E-04 |  | **14** | **17575561** | 3.50E-05 | 2.79E-05 | 6.50E-05 |
| 29 | 15928077 | 3.47E-04 | 2.92E-04 | 4.79E-04 |  | **14** | **17599786** | 3.50E-05 | 2.79E-05 | 6.50E-05 |
| 15 | 28795756 | 3.43E-04 | 3.02E-04 | 4.94E-04 |  | **14** | **17612375** | 3.50E-05 | 2.79E-05 | 6.50E-05 |
| 10 | 56558910 | 3.70E-04 | 3.12E-04 | 5.07E-04 |  | **14** | **17622580** | 3.50E-05 | 2.79E-05 | 6.50E-05 |
| 15 | 28901317 | 3.66E-04 | 3.24E-04 | 5.24E-04 |  | **14** | **17534553** | 4.11E-05 | 3.28E-05 | 7.45E-05 |
| 16 | 71897416 | 3.97E-04 | 3.35E-04 | 5.39E-04 |  | **14** | **17626659** | 4.11E-05 | 3.28E-05 | 7.45E-05 |
| 14 | 18168609 | 4.22E-04 | 3.37E-04 | 8.49E-04 |  | **14** | **17643949** | 4.90E-05 | 3.93E-05 | 8.65E-05 |
| 2 | 99961206 | 4.04E-04 | 3.41E-04 | 5.48E-04 |  | **14** | **33574800** | 5.48E-05 | 4.41E-05 | 9.53E-05 |
| 2 | 99965882 | 4.04E-04 | 3.41E-04 | 5.48E-04 |  | **14** | **17896951** | 5.90E-05 | 4.76E-05 | 1.02E-04 |
| 2 | 99973209 | 4.04E-04 | 3.41E-04 | 5.48E-04 |  | 2 | 96195278 | 6.54E-05 | 5.29E-05 | 1.11E-04 |
| 10 | 72307543 | 4.11E-04 | 3.47E-04 | 5.57E-04 |  | 14 | 34361274 | 7.16E-05 | 5.80E-05 | 1.20E-04 |
| 6 | 74794640 | 4.21E-04 | 3.56E-04 | 5.69E-04 |  | 14 | 34366588 | 7.56E-05 | 6.13E-05 | 1.26E-04 |
| 15 | 21562368 | 4.28E-04 | 3.61E-04 | 5.77E-04 |  | 14 | 17380047 | 1.02E-04 | 8.48E-05 | 1.66E-04 |
| 14 | 18305845 | 4.28E-04 | 3.62E-04 | 5.78E-04 |  | 15 | 28785029 | 1.06E-04 | 9.05E-05 | 1.75E-04 |
| 7 | 22048026 | 7.16E-04 | 3.64E-04 | 1.53E-03 |  | 14 | 33630011 | 1.24E-04 | 1.02E-04 | 1.93E-04 |
| 24 | 42096866 | 4.48E-04 | 3.79E-04 | 6.02E-04 |  | 14 | 34391965 | 1.32E-04 | 1.09E-04 | 2.04E-04 |
| 2 | 33809225 | 1.09E-03 | 3.95E-04 | 1.31E-02 |  | 14 | 17518987 | 1.59E-04 | 1.32E-04 | 2.41E-04 |
| 10 | 57735928 | 4.79E-04 | 4.06E-04 | 6.39E-04 |  | 14 | 36214363 | 2.07E-04 | 1.72E-04 | 3.04E-04 |
| 2 | 32868321 | 5.51E-04 | 4.33E-04 | 1.44E-03 |  | 5 | 6490865 | 1.91E-04 | 1.82E-04 | 3.18E-04 |
| 3 | 117964609 | 4.04E-04 | 4.33E-04 | 6.76E-04 |  | 21 | 48281977 | 2.29E-04 | 1.91E-04 | 3.32E-04 |
| 14 | 15782098 | 5.43E-04 | 4.62E-04 | 7.15E-04 |  | 10 | 56688245 | 2.32E-04 | 1.93E-04 | 3.35E-04 |
| 15 | 28045652 | 5.57E-04 | 4.75E-04 | 7.34E-04 |  | 14 | 18514537 | 2.33E-04 | 1.94E-04 | 3.37E-04 |
| 14 | 18795984 | 5.70E-04 | 4.85E-04 | 7.47E-04 |  | 14 | 18551048 | 2.33E-04 | 1.94E-04 | 3.37E-04 |
| 32 | 117609939 | 4.40E-04 | 4.94E-04 | 7.58E-04 |  | 4 | 28769871 | 2.39E-04 | 1.99E-04 | 3.44E-04 |
| 14 | 82246638 | 5.03E-04 | 4.96E-04 | 7.61E-04 |  | 21 | 48322513 | 2.46E-04 | 2.05E-04 | 3.53E-04 |
| 2 | 99924806 | 5.86E-04 | 4.99E-04 | 7.65E-04 |  | 15 | 27955796 | 2.49E-04 | 2.09E-04 | 3.58E-04 |
| 2 | 99925703 | 5.86E-04 | 4.99E-04 | 7.65E-04 |  | 23 | 40127611 | 2.52E-04 | 2.11E-04 | 3.61E-04 |
| 6 | 76057683 | 5.86E-04 | 4.99E-04 | 7.65E-04 |  | 14 | 34398094 | 2.89E-04 | 2.42E-04 | 4.07E-04 |
|  | | | | | | 15 | 28129429 | 2.53E-04 | 2.44E-04 | 4.10E-04 |
|  |  |  |  |  |  | 1 | 118288481 | 2.87E-04 | 2.44E-04 | 4.11E-04 |
|  |  |  |  |  |  | 15 | 28795756 | 2.78E-04 | 2.50E-04 | 4.18E-04 |
|  |  |  |  |  |  | 15 | 28682409 | 2.73E-04 | 2.53E-04 | 4.23E-04 |
|  |  |  |  |  |  | 21 | 54403433 | 3.11E-04 | 2.76E-04 | 4.57E-04 |
|  |  |  |  |  |  | 21 | 47428967 | 3.22E-04 | 2.78E-04 | 4.60E-04 |
|  |  |  |  |  |  | 21 | 47435380 | 3.22E-04 | 2.78E-04 | 4.60E-04 |
|  |  |  |  |  |  | 21 | 47473673 | 3.22E-04 | 2.78E-04 | 4.60E-04 |
|  |  |  |  |  |  | 14 | 18694622 | 3.34E-04 | 2.81E-04 | 4.63E-04 |
|  |  |  |  |  |  | 29 | 15928077 | 3.47E-04 | 2.92E-04 | 4.79E-04 |
|  |  |  |  |  |  | 10 | 56558910 | 3.70E-04 | 3.12E-04 | 5.07E-04 |
|  |  |  |  |  |  | 5 | 30018379 | 2.71E-04 | 3.21E-04 | 5.20E-04 |
|  |  |  |  |  |  | 15 | 28901317 | 3.67E-04 | 3.24E-04 | 5.24E-04 |
|  |  |  |  |  |  | 16 | 71850677 | 3.97E-04 | 3.35E-04 | 5.39E-04 |
|  |  |  |  |  |  | 16 | 71870410 | 3.97E-04 | 3.35E-04 | 5.39E-04 |
|  |  |  |  |  |  | 16 | 71894122 | 3.97E-04 | 3.35E-04 | 5.39E-04 |
|  |  |  |  |  |  | 16 | 71897416 | 3.97E-04 | 3.35E-04 | 5.39E-04 |
|  |  |  |  |  |  | 2 | 99961206 | 4.04E-04 | 3.41E-04 | 5.48E-04 |
|  |  |  |  |  |  | 2 | 99965882 | 4.04E-04 | 3.41E-04 | 5.48E-04 |
|  |  |  |  |  |  | 2 | 99973209 | 4.04E-04 | 3.41E-04 | 5.48E-04 |
|  |  |  |  |  |  | 14 | 18168609 | 3.99E-04 | 3.46E-04 | 8.49E-04 |
|  |  |  |  |  |  | 10 | 72307543 | 4.11E-04 | 3.47E-04 | 5.57E-04 |
|  |  |  |  |  |  | 10 | 72330195 | 4.11E-04 | 3.47E-04 | 5.57E-04 |
|  |  |  |  |  |  | 10 | 72398954 | 4.11E-04 | 3.47E-04 | 5.57E-04 |
|  |  |  |  |  |  | 2 | 56714041 | 2.08E-04 | 3.60E-04 | 5.75E-04 |
|  |  |  |  |  |  | 15 | 21562368 | 4.28E-04 | 3.61E-04 | 5.77E-04 |
|  |  |  |  |  |  | 14 | 18305845 | 4.27E-04 | 3.62E-04 | 5.78E-04 |
|  |  |  |  |  |  | 1 | 122964231 | 4.36E-04 | 3.69E-04 | 5.87E-04 |
|  |  |  |  |  |  | 16 | 69647723 | 4.47E-04 | 3.78E-04 | 6.00E-04 |
|  |  |  |  |  |  | 24 | 42096866 | 4.48E-04 | 3.79E-04 | 6.02E-04 |
|  |  |  |  |  |  | 2 | 32868321 | 5.52E-04 | 3.94E-04 | 1.44E-03 |
|  |  |  |  |  |  | 10 | 57735928 | 4.79E-04 | 4.06E-04 | 6.39E-04 |
|  |  |  |  |  |  | 14 | 36589147 | 4.90E-04 | 4.16E-04 | 6.52E-04 |
|  |  |  |  |  |  | 6 | 74794640 | 5.01E-04 | 4.25E-04 | 6.65E-04 |
|  |  |  |  |  |  | 16 | 72539868 | 5.09E-04 | 4.32E-04 | 6.75E-04 |
|  |  |  |  |  |  | 3 | 117964609 | 4.14E-04 | 4.33E-04 | 6.76E-04 |
|  |  |  |  |  |  | 24 | 34921668 | 5.18E-04 | 4.40E-04 | 6.85E-04 |
|  |  |  |  |  |  | 7 | 76674069 | 3.53E-04 | 4.42E-04 | 6.88E-04 |
|  |  |  |  |  |  | 14 | 15782098 | 5.43E-04 | 4.62E-04 | 7.15E-04 |
|  |  |  |  |  |  | 1 | 119519463 | 5.47E-04 | 4.65E-04 | 7.19E-04 |
|  |  |  |  |  |  | 15 | 28045652 | 5.60E-04 | 4.75E-04 | 7.34E-04 |
|  |  |  |  |  |  | 14 | 18795984 | 5.70E-04 | 4.85E-04 | 7.47E-04 |
|  |  |  |  |  |  | 32 | 117609939 | 4.22E-04 | 4.94E-04 | 7.58E-04 |
|  |  |  |  |  |  | 14 | 82246638 | 5.00E-04 | 4.96E-04 | 7.61E-04 |
|  |  |  |  |  |  | 2 | 99924806 | 5.86E-04 | 4.99E-04 | 7.65E-04 |
|  |  |  |  |  |  | 2 | 99925703 | 5.86E-04 | 4.99E-04 | 7.65E-04 |
|  |  |  |  |  |  | 6 | 76057683 | 5.86E-04 | 4.99E-04 | 7.65E-04 |

**Table S4:** Summary of variants by type and region. Some variants were predicted to have more than one possible effect, so were assigned to more than one type or region.

| **TYPE** | | | **REGION** | | |
| --- | --- | --- | --- | --- | --- |
| **Type** | **Number** | **Percent** | **Type** | **Number** | **Percent** |
| codon change + codon deletion | 119 | 0.001 | downstream | 802,140 | 4.65 |
| codon change + codon insertion | 66 | <0.001 | exon | 170,216 | 0.99 |
| codon deletion | 155 | 0.001 | intergenic | 9,741,652 | 56.43 |
| codon insertion | 114 | 0.001 | intron | 4,438,806 | 25.71 |
| downstream | 802,140 | 4.65 | none | 1,221,763 | 7.08 |
| exon | 20,282 | 0.12 | splice site acceptor | 921 | 0.005 |
| exon deleted | 2 | <0.001 | splice site donor | 1,099 | 0.006 |
| frame shift | 6,946 | 0.04 | upstream | 867,574 | 5.03 |
| intergenic | 9,741,652 | 56.43 | 3’UTR | 13,024 | 0.08 |
| intragenic | 101 | 0.001 | 5’UTR | 7,472 | 0.04 |
| intron | 4,438,806 | 25.71 |  | | |
| none | 1,221,763 | 7.08 |  |  |  |
| nonsynonymous coding | 56,668 | 0.33 |  |  |  |
| nonsynonymous start | 16 | <0.001 |  |  |  |
| splice site acceptor | 921 | 0.005 |  |  |  |
| splice site donor | 1,099 | 0.006 |  |  |  |
| start gained | 746 | 0.004 |  |  |  |
| start lost | 58 | <0.001 |  |  |  |
| stop gained | 596 | 0.003 |  |  |  |
| stop lost | 40 | <0.001 |  |  |  |
| synonymous coding | 85,097 | 0.49 |  |  |  |
| synonymous start | 3 | <0.001 |  |  |  |
| synonymous stop | 54 | <0.001 |  |  |  |
| upstream | 867,574 | 5.03 |  |  |  |
| 3’UTR | 13,024 | 0.08 |  |  |  |
| 5’UTR | 6,726 | 0.04 |  |  |  |

**Table S5:** Regions of interest for which detailed annotation of SNPs was performed. Previously reported regions marked in **bold** were prioritized because they were associated with tarsal OC (instead of other anatomical locations), discovered in Standardbreds, and/or reported in multiple studies. GWAS = genome-wide association study; Chr = chromosome.

| Regions of interest from discovery cohort GWAS | | Previously reported regions of association | |
| --- | --- | --- | --- |
| **Chr** | **Region** | **Chr** | **Region** |
| 1 | 117-119Mb | **1** | **139-140Mb^3^** |
| 2 | 70-78Mb | **2** | **15-34Mb^1,7^** |
| 2 | 98-100Mb | **3** | **88-114Mb^3,4,5^** |
| 6 | 24-26Mb | 4 | 3-40Mb^5,7^ |
| 10 | 55-57Mb | **4** | **56-60Mb^7^** |
| 14 | 15-19Mb | 4 | 76-78Mb^3^ |
| 14 | 32-39Mb | 5 | 41-43Mb^2,3,7^ |
| 15 | 27-29Mb | **5** | **76-92Mb^3^** |
| 16 | 49-52Mb | 9 | 17-19Mb^3^ |
| 21 | 47-55Mb | 10 | 59-61Mb^3^ |
|  | | 10 | 79-81Mb^3^ |
|  |  | 13 | 0-13Mb^4^ |
|  |  | **14** | **67-79Mb^4^** |
|  |  | 15 | 75-90Mb^4^ |
|  |  | **16** | **6-25Mb^7^** |
|  |  | **16** | **33-43Mb^7^** |
|  |  | **18** | **35-47Mb^5,6^** |
|  |  | 18 | 58-59Mb^3^ |
|  |  | **18** | **74-83Mb^2,7^** |
|  |  | **21** | **5-17Mb^7^** |
|  |  | 27 | 38-39Mb^3^ |
|  |  | 28 | 41-43Mb^3^ |

^1^ Dierks et al. *Anim Genet* 41 Suppl. 2:87-90, 2010.

^2^ Lampe et al. *Anim Genet* 40:553-555, 2009.

^3^ Lykkjen et al. *Anim Genet* 41 Suppl. 2:111-120, 2010.

^4^ Teyssédre et al. *J Anim Sci* 90:45-53, 2012.

^5^ Corbin et al. *Mamm Genome* 23:294-303, 2012.

^6^ Wittwer et al. *J Hered* 100:481-486, 2009.

^7^ unpublished thesis, University of Hannover

**Table S6:** Putative risk variants for OC that were selected for inclusion in the custom Sequenom genotyping assay (n = 240). SNPs were multiplexed in groups of 48 in 5 separate wells.

| **WELL 1** | |  | **WELL 2** | |  | **WELL 3** | |  | **WELL 4** | |  | **WELL 5** | |
| --- | --- | --- | --- | --- | --- | --- | --- | --- | --- | --- | --- | --- | --- |
| **Chr** | **bp** |  | **Chr** | **bp** |  | **Chr** | **bp** |  | **Chr** | **bp** |  | **Chr** | **bp** |
| 2 | 98927433 |  | 14 | 37348824 |  | 1 | 118796012 |  | 21 | 50321052 |  | 14 | 36243090 |
| 18 | 75969689 |  | 16 | 43467550 |  | 14 | 34803961 |  | 21 | 52586460 |  | 21 | 4512138 |
| 18 | 77703956 |  | 4 | 6190928 |  | 1 | 118846185 |  | 10 | 57468165 |  | 2 | 28136111 |
| 18 | 75780078 |  | 16 | 23943994 |  | 14 | 16802524 |  | 14 | 35652608 |  | 16 | 41459922 |
| 21 | 4598516 |  | 16 | 34073553 |  | 14 | 18323534 |  | 14 | 36115804 |  | 3 | 89027561 |
| 2 | 29591774 |  | 10 | 59079917 |  | 14 | 36238870 |  | 14 | 37213468 |  | 2 | 33902705 |
| 18 | 77894560 |  | 1 | 118105257 |  | 16 | 43285189 |  | 14 | 35832191 |  | 2 | 30971463 |
| 18 | 75504584 |  | 14 | 35749215 |  | 14 | 18757945 |  | 16 | 36711709 |  | 14 | 36975745 |
| 2 | 32190656 |  | 18 | 39910627 |  | 14 | 35480068 |  | 14 | 38496150 |  | 16 | 39306626 |
| 16 | 34620840 |  | 16 | 14358731 |  | 14 | 36270564 |  | 10 | 59685445 |  | 2 | 23390833 |
| 18 | 77725062 |  | 16 | 34954141 |  | 2 | 31863561 |  | 14 | 67874878 |  | 10 | 80739334 |
| 14 | 15545589 |  | 10 | 80792903 |  | 4 | 5924012 |  | 21 | 4707138 |  | 14 | 35160061 |
| 21 | 4800562 |  | 16 | 20892756 |  | 14 | 36627081 |  | 10 | 57209370 |  | 21 | 51353146 |
| 14 | 35338969 |  | 21 | 4800528 |  | 16 | 41794782 |  | 1 | 117892759 |  | 1 | 140205123 |
| 16 | 41804869 |  | 16 | 41787035 |  | 10 | 56727782 |  | 14 | 38120832 |  | 14 | 36174501 |
| 2 | 32641671 |  | 18 | 44859933 |  | 1 | 117500403 |  | 14 | 35359271 |  | 14 | 36078935 |
| 18 | 76006633 |  | 5 | 77353904 |  | 14 | 18198820 |  | 10 | 57134088 |  | 16 | 41794953 |
| 2 | 18190159 |  | 10 | 55605051 |  | 1 | 139944477 |  | 14 | 38261144 |  | 21 | 51448245 |
| 16 | 17404735 |  | 5 | 77536297 |  | 1 | 118324956 |  | 1 | 118771557 |  | 14 | 35581792 |
| 14 | 18322233 |  | 14 | 38011286 |  | 21 | 52594185 |  | 14 | 38237645 |  | 16 | 38384099 |
| 14 | 38234471 |  | 3 | 88076689 |  | 14 | 16857276 |  | 10 | 57167028 |  | 1 | 139695746 |
| 21 | 7509248 |  | 18 | 40807803 |  | 14 | 17365436 |  | 14 | 35042619 |  | 21 | 49216451 |
| 16 | 20901110 |  | 14 | 36239254 |  | 14 | 37127327 |  | 14 | 16830511 |  | 2 | 19959258 |
| 16 | 41546606 |  | 10 | 80690472 |  | 14 | 34156670 |  | 14 | 36226740 |  | 21 | 4898282 |
| 21 | 6300122 |  | 1 | 139375281 |  | 14 | 33691422 |  | 14 | 37321714 |  | 14 | 72031059 |
| 18 | 40478429 |  | 21 | 4515908 |  | 14 | 36098913 |  | 10 | 56817838 |  | 14 | 17882983 |
| 14 | 18034557 |  | 10 | 59873648 |  | 14 | 33820804 |  | 1 | 118401125 |  | 14 | 37568111 |
| 21 | 51305453 |  | 1 | 139685697 |  | 21 | 53591449 |  | 14 | 34391965 |  | 21 | 50383063 |
| 18 | 75992716 |  | 14 | 36012505 |  | 14 | 35750986 |  | 14 | 35638840 |  | 21 | 6611863 |
| 14 | 16782922 |  | 14 | 34929440 |  | 14 | 35353077 |  | 14 | 16776824 |  | 14 | 38224509 |
| 16 | 12795866 |  | 1 | 117511240 |  | 21 | 51408645 |  | 14 | 18528304 |  | 21 | 50238955 |
| 14 | 72832737 |  | 21 | 52365784 |  | 2 | 99999249 |  | 1 | 117907704 |  | 14 | 38578258 |
| 1 | 117899604 |  | 1 | 118839637 |  | 2 | 30472121 |  | 21 | 53794214 |  | 14 | 38157667 |
| 14 | 35710575 |  | 14 | 33108459 |  | 21 | 48664783 |  | 1 | 140238061 |  | 14 | 38297106 |
| 18 | 75187666 |  | 18 | 46490552 |  | 21 | 49368721 |  | 14 | 38421896 |  | 21 | 53288223 |
| 5 | 77353905 |  | 18 | 42386473 |  | 14 | 71698112 |  | 14 | 36386541 |  | 14 | 35110220 |
| 2 | 30971496 |  | 3 | 107352236 |  | 14 | 35363931 |  | 10 | 55518157 |  | 14 | 36321021 |
| 5 | 78709303 |  | 10 | 57350466 |  | 14 | 35727280 |  | 14 | 18209193 |  | 21 | 50250540 |
| 14 | 35733635 |  | 14 | 16840478 |  | 14 | 17825358 |  | 14 | 72832742 |  | 10 | 56789024 |
| 14 | 38640879 |  | 21 | 53928489 |  | 21 | 6590487 |  | 14 | 70569943 |  | 14 | 35796385 |
| 2 | 98925499 |  | 21 | 50348105 |  | 21 | 49882816 |  | 14 | 16857186 |  | 14 | 36231214 |
| 14 | 73999237 |  | 1 | 117508428 |  | 14 | 18059791 |  | 14 | 36762857 |  | 14 | 35713816 |
| 14 | 16538670 |  | 1 | 117896863 |  | 14 | 17829592 |  | 16 | 38404778 |  | 1 | 118293860 |
| 21 | 51402003 |  | 10 | 55512346 |  | 14 | 34520718 |  | 14 | 38740729 |  | 14 | 34945056 |
| 14 | 35681098 |  | 16 | 20876274 |  | 2 | 99336592 |  | 14 | 18029925 |  | 10 | 55657837 |
| 21 | 53443537 |  | 14 | 33234861 |  | 18 | 39195340 |  | 14 | 36302342 |  | 1 | 117503692 |
| 21 | 49950751 |  | 10 | 57303131 |  | 14 | 16782779 |  | 14 | 16854653 |  | 14 | 34256372 |
| 14 | 37281732 |  | 14 | 34940505 |  | 1 | 117545952 |  | 21 | 51325270 |  | 14 | 32504217 |

**Table S7:** Frequency of alternate allele in cases and controls for each SNP in the Sequenom platform that genotyped successfully in the discovery or validation populations. # CHR = number of chromosomes upon which allele frequency was calculated.

|  | **Discovery Cohort** | | | | **Validation Cohort** | | | |
| --- | --- | --- | --- | --- | --- | --- | --- | --- |
| SNP | Controls | # CHR | Cases | # CHR | Controls | # CHR | Cases | # CHR |
| 1-117500403_A | 0.157534 | 146 | 0.255814 | 86 | 0 | 144 | 0 | 92 |
| 1-117503692_A | 0.107955 | 176 | 0.113636 | 132 | 0.070513 | 156 | 0.033333 | 120 |
| 1-117508428_0 | 0 | 218 | 0 | 142 | 0 | 158 | 0 | 120 |
| 1-117511240_0 | 0 | 218 | 0 | 142 | 0 | 158 | 0 | 120 |
| 1-117545952_T | 0.283019 | 212 | 0.333333 | 138 | 0.201299 | 154 | 0.241379 | 116 |
| 1-117892759_G | 0.056075 | 214 | 0.071429 | 140 | 0.064103 | 156 | 0.026316 | 114 |
| 1-117896863_C | 0.282407 | 216 | 0.333333 | 138 | 0.208861 | 158 | 0.233333 | 120 |
| 1-117899604_G | 0.275701 | 214 | 0.264286 | 140 | 0.107595 | 158 | 0.108333 | 120 |
| 1-117907704_T | 0.046729 | 214 | 0.071429 | 140 | 0.064103 | 156 | 0.025862 | 116 |
| 1-118105257_T | 0.277778 | 216 | 0.257143 | 140 | 0.107595 | 158 | 0.108333 | 120 |
| 1-118293860_T | 0.303191 | 188 | 0.272727 | 110 | 0.209677 | 124 | 0.090909 | 88 |
| 1-118324956_T | 0.273585 | 212 | 0.268116 | 138 | 0.114583 | 96 | 0.078947 | 76 |
| 1-118401125_C | 0.481308 | 214 | 0.392857 | 140 | 0.531646 | 158 | 0.533898 | 118 |
| 1-118771557_A | 0.485981 | 214 | 0.391304 | 138 | 0.556962 | 158 | 0.567797 | 118 |
| 1-118796012_G | 0.476415 | 212 | 0.507246 | 138 | 0.344156 | 154 | 0.318966 | 116 |
| 1-118839637_T | 0.416667 | 216 | 0.456522 | 138 | 0.664557 | 158 | 0.6 | 120 |
| 1-118846185_G | 0.436893 | 206 | 0.398551 | 138 | 0.19403 | 134 | 0.294118 | 102 |
| 1-139375281_T | 0.208333 | 216 | 0.185714 | 140 | 0.265823 | 158 | 0.358333 | 120 |
| 1-139685697_T | 0.296296 | 216 | 0.342857 | 140 | 0.398734 | 158 | 0.225 | 120 |
| 1-139695746_G | 0.19802 | 202 | 0.213235 | 136 | 0.25 | 156 | 0.166667 | 120 |
| 1-139944477_T | 0.278302 | 212 | 0.318841 | 138 | 0.363636 | 154 | 0.25 | 116 |
| 1-140205123_G | 0.282178 | 202 | 0.314286 | 140 | 0.396104 | 154 | 0.208333 | 120 |
| 1-140238061_T | 0.303738 | 214 | 0.214286 | 140 | 0.24359 | 156 | 0.336207 | 116 |
| 2-18190159_G | 0.401869 | 214 | 0.335714 | 140 | 0.310127 | 158 | 0.35 | 120 |
| 2-19959258_C | 0.22549 | 204 | 0.246377 | 138 | 0.410256 | 156 | 0.4 | 120 |
| 2-23390833_A | 0.183168 | 202 | 0.228571 | 140 | 0.076923 | 156 | 0.075 | 120 |
| 2-28136111_C | 0.474747 | 198 | 0.441176 | 136 | 0.493506 | 154 | 0.542373 | 118 |
| 2-29591774_C | 0.308411 | 214 | 0.288732 | 142 | 0.132911 | 158 | 0.2 | 120 |
| 2-30472121_G | 0.419811 | 212 | 0.376812 | 138 | 0.559211 | 152 | 0.5 | 116 |
| 2-30971463_C | 0.122449 | 196 | 0.068182 | 132 | 0.160256 | 156 | 0.208333 | 120 |
| 2-30971496_A | 0.453271 | 214 | 0.352941 | 136 | 0.538462 | 156 | 0.483333 | 120 |
| 2-31863561_T | 0.509524 | 210 | 0.492537 | 134 | 0.487013 | 154 | 0.482759 | 116 |
| 2-32190656_A | 0.481308 | 214 | 0.492754 | 138 | 0.487342 | 158 | 0.533333 | 120 |
| 2-32641671_C | 0.364486 | 214 | 0.4 | 140 | 0.348101 | 158 | 0.383333 | 120 |
| 2-33902705_C | 0.336634 | 202 | 0.413043 | 138 | 0.538462 | 156 | 0.55 | 120 |
| 2-98927433_A | 0.189815 | 216 | 0.235714 | 140 | 0.21519 | 158 | 0.25 | 120 |
| 2-99336592_T | 0.283019 | 212 | 0.355072 | 138 | 0.350649 | 154 | 0.327586 | 116 |
| 2-99999249_A | 0.146226 | 212 | 0.057971 | 138 | 0.103896 | 154 | 0.12069 | 116 |
| 3-88076689_T | 0.305556 | 216 | 0.335714 | 140 | 0.240506 | 158 | 0.283333 | 120 |
| 3-89027561_T | 0.435 | 200 | 0.507042 | 142 | 0.532051 | 156 | 0.458333 | 120 |
| 3-107352236_G | 0.439815 | 216 | 0.371429 | 140 | 0.544304 | 158 | 0.325 | 120 |
| 4-5924012_G | 0.254717 | 212 | 0.314286 | 140 | 0.266234 | 154 | 0.456897 | 116 |
| 4-6190928_C | 0.425926 | 216 | 0.376812 | 138 | 0.360759 | 158 | 0.275 | 120 |
| 5-77353904_0 | 0 | 216 | 0 | 140 | 0 | 158 | 0 | 120 |
| 5-77353905_0 | 0 | 198 | 0 | 126 | 0 | 158 | 0 | 120 |
| 5-77536297_T | 0.134259 | 216 | 0.112676 | 142 | 0.170886 | 158 | 0.083333 | 120 |
| 5-78709303_C | 0.28972 | 214 | 0.328571 | 140 | 0.202532 | 158 | 0.333333 | 120 |
| 10-55512346_C | 0.027778 | 216 | 0.064286 | 140 | 0 | 158 | 0 | 120 |
| 10-55518157_A | 0.168224 | 214 | 0.149254 | 134 | 0.198718 | 156 | 0.196429 | 112 |
| 10-55605051_A | 0.074074 | 216 | 0.166667 | 138 | 0.037975 | 158 | 0.008333 | 120 |
| 10-55657837_A | 0.040404 | 198 | 0.097015 | 134 | 0 | 156 | 0 | 120 |
| 10-56727782_A | 0.5 | 212 | 0.5 | 140 | 0.5 | 154 | 0.517241 | 116 |
| 10-56789024_A | 0.311224 | 196 | 0.215385 | 130 | 0.434211 | 152 | 0.4 | 120 |
| 10-56817838_T | 0.045872 | 218 | 0.098592 | 142 | 0.044304 | 158 | 0.008621 | 116 |
| 10-57134088_G | 0.443925 | 214 | 0.401408 | 142 | 0.487179 | 156 | 0.415254 | 118 |
| 10-57167028_G | 0.375 | 72 | 0.326923 | 52 | 0.037037 | 54 | 0.055556 | 36 |
| 10-57209370_A | 0.140187 | 214 | 0.181159 | 138 | 0.107595 | 158 | 0.033898 | 118 |
| 10-57303131_C | 0.365741 | 216 | 0.464286 | 140 | 0.21519 | 158 | 0.125 | 120 |
| 10-57350466_T | 0.407407 | 216 | 0.543478 | 138 | 0.291139 | 158 | 0.225 | 120 |
| 10-57468165_G | 0.459302 | 172 | 0.387097 | 124 | 0.22449 | 98 | 0.196429 | 56 |
| 10-59079917_A | 0.199074 | 216 | 0.242857 | 140 | 0.101266 | 158 | 0.1 | 120 |
| 10-59685445_0 | 0 | 214 | 0 | 140 | 0 | 158 | 0 | 118 |
| 10-59873648_T | 0.111111 | 216 | 0.152174 | 138 | 0.06962 | 158 | 0.05 | 120 |
| 10-80690472_T | 0.333333 | 216 | 0.326087 | 138 | 0.158228 | 158 | 0.116667 | 120 |
| 10-80739334_C | 0.14 | 200 | 0.123077 | 130 | 0.136364 | 154 | 0.137931 | 116 |
| 10-80792903_C | 0.383178 | 214 | 0.404412 | 136 | 0.550633 | 158 | 0.475 | 120 |
| 14-15545589_0 | 0 | 216 | 0 | 142 | 0 | 158 | 0 | 120 |
| 14-16538670_A | 0.331776 | 214 | 0.25 | 140 | 0.506329 | 158 | 0.425 | 120 |
| 14-16776824_G | 0.232143 | 168 | 0.327586 | 116 | 0.164179 | 134 | 0.353659 | 82 |
| 14-16782779_A | 0.157407 | 216 | 0.105634 | 142 | 0.328947 | 152 | 0.359649 | 114 |
| 14-16782922_G | 0.263889 | 216 | 0.157143 | 140 | 0.373418 | 158 | 0.333333 | 120 |
| 14-16802524_C | 0.291667 | 216 | 0.25 | 132 | 0.263158 | 152 | 0.258621 | 116 |
| 14-16830511_C | 0.271028 | 214 | 0.335714 | 140 | 0.183544 | 158 | 0.318966 | 116 |
| 14-16840478_G | 0.306604 | 212 | 0.365672 | 134 | 0.190789 | 152 | 0.322034 | 118 |
| 14-16854653_C | 0.215596 | 218 | 0.133803 | 142 | 0.410256 | 156 | 0.37931 | 116 |
| 14-16857186_0 | 0 | 214 | 0 | 138 | 0 | 156 | 0 | 116 |
| 14-16857276_0 | 0 | 212 | 0 | 138 | 0 | 154 | 0 | 116 |
| 14-17365436_A | 0.458333 | 216 | 0.359155 | 142 | 0.493506 | 154 | 0.5 | 116 |
| 14-17825358_C | 0.45283 | 212 | 0.5 | 138 | 0.309211 | 152 | 0.37069 | 116 |
| 14-17829592_A | 0.457143 | 210 | 0.5 | 138 | 0.276923 | 130 | 0.387755 | 98 |
| 14-17882983_T | 0.168317 | 202 | 0.095588 | 136 | 0.435897 | 156 | 0.425 | 120 |
| 14-18029925_G | 0.158879 | 214 | 0.092857 | 140 | 0.386076 | 158 | 0.305085 | 118 |
| 14-18034557_T | 0.182243 | 214 | 0.112676 | 142 | 0.398734 | 158 | 0.35 | 120 |
| 14-18059791_T | 0.183962 | 212 | 0.114286 | 140 | 0.378378 | 148 | 0.350877 | 114 |
| 14-18198820_C | 0.25 | 212 | 0.294118 | 136 | 0.227273 | 154 | 0.396552 | 116 |
| 14-18209193_T | 0.242991 | 214 | 0.164286 | 140 | 0.423077 | 156 | 0.387931 | 116 |
| 14-18322233_G | 0.406542 | 214 | 0.405797 | 138 | 0.436709 | 158 | 0.475 | 120 |
| 14-18323534_T | 0.377358 | 212 | 0.407143 | 140 | 0.383117 | 154 | 0.482759 | 116 |
| 14-18528304_A | 0.21028 | 214 | 0.314286 | 140 | 0.246835 | 158 | 0.336207 | 116 |
| 14-18757945_T | 0.171569 | 204 | 0.253846 | 130 | 0.123377 | 154 | 0.206897 | 116 |
| 14-32504217_T | 0.380208 | 192 | 0.420635 | 126 | 0.351351 | 148 | 0.392157 | 102 |
| 14-33108459_C | 0.439252 | 214 | 0.485714 | 140 | 0.658228 | 158 | 0.65 | 120 |
| 14-33234861_C | 0.127551 | 196 | 0.156716 | 134 | 0.14557 | 158 | 0.15 | 120 |
| 14-33691422_C | 0.004808 | 208 | 0.007353 | 136 | 0 | 144 | 0.018182 | 110 |
| 14-33820804_A | 0.278302 | 212 | 0.221429 | 140 | 0.175325 | 154 | 0.206897 | 116 |
| 14-34156670_G | 0.278302 | 212 | 0.224638 | 138 | 0.153333 | 150 | 0.205357 | 112 |
| 14-34256372_G | 0.282178 | 202 | 0.235294 | 136 | 0.160256 | 156 | 0.216667 | 120 |
| 14-34391965_C | 0.079439 | 214 | 0.195652 | 138 | 0.11039 | 154 | 0.113208 | 106 |
| 14-34520718_A | 0.273585 | 212 | 0.231884 | 138 | 0.155844 | 154 | 0.224138 | 116 |
| 14-34803961_T | 0.04717 | 212 | 0.114286 | 140 | 0.071429 | 154 | 0.094828 | 116 |
| 14-34929440_A | 0.310185 | 216 | 0.282609 | 138 | 0.35443 | 158 | 0.3 | 120 |
| 14-34940505_A | 0.310185 | 216 | 0.292857 | 140 | 0.35443 | 158 | 0.3 | 120 |
| 14-34945056_G | 0.270408 | 196 | 0.204545 | 132 | 0.307692 | 156 | 0.266667 | 120 |
| 14-35042619_C | 0.383178 | 214 | 0.405797 | 138 | 0.537975 | 158 | 0.533898 | 118 |
| 14-35160061_C | 0.377451 | 204 | 0.362319 | 138 | 0.285714 | 154 | 0.283333 | 120 |
| 14-35338969_A | 0.258065 | 62 | 0.1875 | 32 | Did not genotype successfully | | | |
| 14-35353077_G | 0.396226 | 212 | 0.413043 | 138 | 0.507353 | 136 | 0.489583 | 96 |
| 14-35359271_G | 0.103774 | 212 | 0.088235 | 136 | 0.221519 | 158 | 0.163793 | 116 |
| 14-35363931_C | 0.04717 | 212 | 0.123188 | 138 | 0.071429 | 154 | 0.094828 | 116 |
| 14-35480068_A | 0.504717 | 212 | 0.478261 | 138 | 0.584416 | 154 | 0.594828 | 116 |
| 14-35581792_C | 0.397059 | 204 | 0.407143 | 140 | 0.519481 | 154 | 0.5 | 120 |
| 14-35638840_G | 0.397196 | 214 | 0.407143 | 140 | 0.518987 | 158 | 0.5 | 118 |
| 14-35652608_G | 0.399083 | 218 | 0.326087 | 138 | 0.132911 | 158 | 0.177966 | 118 |
| 14-35681098_A | 0.397196 | 214 | 0.421429 | 140 | 0.518987 | 158 | 0.5 | 120 |
| 14-35710575_C | 0.398148 | 216 | 0.407143 | 140 | 0.518987 | 158 | 0.5 | 120 |
| 14-35713816_G | 0.40404 | 198 | 0.415385 | 130 | 0.519481 | 154 | 0.5 | 120 |
| 14-35727280_T | 0.364486 | 214 | 0.285714 | 140 | 0.253247 | 154 | 0.241379 | 116 |
| 14-35733635_T | 0.101852 | 216 | 0.091549 | 142 | 0.221519 | 158 | 0.158333 | 120 |
| 14-35749215_0 | 0 | 216 | 0 | 136 | 0 | 158 | 0 | 120 |
| 14-35750986_A | 0.396226 | 212 | 0.413043 | 138 | 0.525974 | 154 | 0.517241 | 116 |
| 14-35796385_A | 0.416667 | 180 | 0.460784 | 102 | 0.520548 | 146 | 0.531915 | 94 |
| 14-35832191_G | 0.397196 | 214 | 0.407143 | 140 | 0.518987 | 158 | 0.5 | 118 |
| 14-36078935_G | 0.10396 | 202 | 0.085714 | 140 | 0.214286 | 154 | 0.158333 | 120 |
| 14-36098913_T | 0.082353 | 170 | 0.033333 | 120 | 0 | 152 | 0 | 116 |
| 14-36115804_0 | 0 | 216 | 0 | 142 | 0 | 158 | 0 | 118 |
| 14-36174501_0 | 0 | 204 | 0 | 142 | 0 | 156 | 0 | 120 |
| 14-36226740_C | 0.420561 | 214 | 0.414286 | 140 | 0.537975 | 158 | 0.508475 | 118 |
| 14-36231214_G | 0.108911 | 202 | 0.094203 | 138 | 0.217949 | 156 | 0.158333 | 120 |
| 14-36238870_G | 0.033019 | 212 | 0.014493 | 138 | 0.090909 | 154 | 0.043103 | 116 |
| 14-36239254_G | Did not genotype successfully | | | | 0.03125 | 128 | 0.023256 | 86 |
| 14-36243090_G | 0.015152 | 198 | 0.053846 | 130 | 0.102564 | 156 | 0.083333 | 120 |
| 14-36270564_G | 0.423077 | 182 | 0.423729 | 118 | 0.490741 | 108 | 0.583333 | 84 |
| 14-36302342_A | 0.423529 | 170 | 0.457447 | 94 | 0.536765 | 136 | 0.56383 | 94 |
| 14-36321021_G | 0.405941 | 202 | 0.407143 | 140 | 0.525641 | 156 | 0.5 | 120 |
| 14-36386541_C | 0.407407 | 216 | 0.407143 | 140 | 0.525316 | 158 | 0.5 | 116 |
| 14-36627081_C | 0.433962 | 212 | 0.449275 | 138 | 0.545455 | 154 | 0.543103 | 116 |
| 14-36762857_0 | 0 | 88 | 0 | 42 | 0 | 62 | 0 | 34 |
| 14-36975745_G | 0.28 | 200 | 0.282609 | 138 | 0.525641 | 156 | 0.483333 | 120 |
| 14-37127327_C | 0.04717 | 212 | 0.114286 | 140 | 0.064935 | 154 | 0.086207 | 116 |
| 14-37213468_G | 0.208738 | 206 | 0.358209 | 134 | 0.33871 | 124 | 0.333333 | 102 |
| 14-37281732_T | 0.257009 | 214 | 0.23913 | 138 | 0.158228 | 158 | 0.258333 | 120 |
| 14-37321714_0 | 0 | 214 | 0 | 140 | 0 | 158 | 0 | 118 |
| 14-37348824_0 | 0 | 216 | 0 | 140 | 0 | 158 | 0 | 120 |
| 14-37568111_G | 0.227723 | 202 | 0.23913 | 138 | 0.391026 | 156 | 0.333333 | 120 |
| 14-38011286_0 | 0 | 216 | 0 | 140 | 0 | 158 | 0 | 120 |
| 14-38120832_0 | 0 | 214 | 0 | 140 | 0 | 156 | 0 | 118 |
| 14-38157667_G | 0.5 | 202 | 0.5 | 136 | 0.5 | 156 | 0.5 | 120 |
| 14-38224509_0 | 0 | 200 | 0 | 130 | 0 | 154 | 0 | 120 |
| 14-38234471_T | 0.163551 | 214 | 0.15942 | 138 | 0.294872 | 156 | 0.225 | 120 |
| 14-38237645_C | 0.098131 | 214 | 0.086957 | 138 | 0.221519 | 158 | 0.161017 | 118 |
| 14-38261144_T | 0.12963 | 216 | 0.128571 | 140 | 0.025316 | 158 | 0.042373 | 118 |
| 14-38297106_T | 0.475 | 160 | 0.475 | 120 | 0.16129 | 124 | 0.22619 | 84 |
| 14-38421896_G | 0.267606 | 142 | 0.277778 | 90 | Did not genotype successfully | | | |
| 14-38496150_C | 0.205607 | 214 | 0.135714 | 140 | 0.107595 | 158 | 0.043103 | 116 |
| 14-38578258_A | 0.138614 | 202 | 0.126761 | 142 | 0.032051 | 156 | 0.041667 | 120 |
| 14-38640879_C | 0.102804 | 214 | 0.107143 | 140 | 0.240506 | 158 | 0.166667 | 120 |
| 14-38740729_T | 0.292453 | 212 | 0.355072 | 138 | 0.462025 | 158 | 0.465517 | 116 |
| 14-67874878_G | 0.093458 | 214 | 0.057143 | 140 | 0.202532 | 158 | 0.189655 | 116 |
| 14-70569943_G | 0.313084 | 214 | 0.333333 | 138 | 0.266667 | 150 | 0.342593 | 108 |
| 14-71698112_G | 0.29717 | 212 | 0.272059 | 136 | 0.361842 | 152 | 0.285714 | 112 |
| 14-72031059_T | 0.29703 | 202 | 0.268116 | 138 | 0.358974 | 156 | 0.3 | 120 |
| 14-72832737_0 | 0 | 214 | 0 | 138 | 0 | 158 | 0 | 120 |
| 14-72832742_0 | 0 | 214 | 0 | 140 | 0 | 158 | 0 | 116 |
| 14-73999237_G | 0.266355 | 214 | 0.242857 | 140 | 0.303797 | 158 | 0.3 | 120 |
| 16-12795866_G | 0.163551 | 214 | 0.157143 | 140 | 0.139241 | 158 | 0.116667 | 120 |
| 16-14358731_T | 0.439815 | 216 | 0.326087 | 138 | 0.405063 | 158 | 0.391667 | 120 |
| 16-17404735_T | 0.313084 | 214 | 0.357143 | 140 | 0.272152 | 158 | 0.291667 | 120 |
| 16-20876274_G | 0.194444 | 216 | 0.157143 | 140 | 0.189873 | 158 | 0.191667 | 120 |
| 16-20892756_C | 0.208333 | 216 | 0.221429 | 140 | 0.107595 | 158 | 0.116667 | 120 |
| 16-20901110_G | 0.21028 | 214 | 0.221429 | 140 | 0.107595 | 158 | 0.116667 | 120 |
| 16-34073553_A | 0.175926 | 216 | 0.147059 | 136 | 0.253165 | 158 | 0.258333 | 120 |
| 16-34620840_G | 0.280374 | 214 | 0.279412 | 136 | 0.35443 | 158 | 0.391667 | 120 |
| 16-34954141_0 | 0 | 10 | 0 | 2 | 0 | 144 | 0 | 114 |
| 16-36711709_T | 0.14486 | 214 | 0.123188 | 138 | 0.09589 | 146 | 0.12766 | 94 |
| 16-38384099_G | 0.19802 | 202 | 0.173913 | 138 | 0.416667 | 156 | 0.475 | 120 |
| 16-38404778_A | 0.226415 | 212 | 0.204225 | 142 | 0.49359 | 156 | 0.508475 | 118 |
| 16-39306626_G | 0.39604 | 202 | 0.405797 | 138 | 0.301282 | 156 | 0.216667 | 120 |
| 16-41459922_G | 0.146739 | 184 | 0.091667 | 120 | 0.415584 | 154 | 0.5 | 118 |
| 16-41546606_C | 0.240741 | 216 | 0.25 | 140 | 0.348101 | 158 | 0.4 | 120 |
| 16-41787035_C | 0.5 | 216 | 0.507246 | 138 | 0.28481 | 158 | 0.216667 | 120 |
| 16-41794782_A | 0.490566 | 212 | 0.507246 | 138 | 0.285714 | 154 | 0.224138 | 116 |
| 16-41794953_C | 0.49505 | 202 | 0.514286 | 140 | 0.279221 | 154 | 0.216667 | 120 |
| 16-41804869_G | 0.5 | 214 | 0.5 | 140 | 0.28481 | 158 | 0.216667 | 120 |
| 16-43285189_C | 0.34434 | 212 | 0.391304 | 138 | 0.545455 | 154 | 0.491379 | 116 |
| 16-43467550_G | 0.083333 | 216 | 0.057971 | 138 | 0.056962 | 158 | 0.05 | 120 |
| 18-39195340_C | 0.226415 | 212 | 0.278571 | 140 | 0.305195 | 154 | 0.318966 | 116 |
| 18-39910627_C | 0.277778 | 216 | 0.352941 | 136 | 0.512658 | 158 | 0.55 | 120 |
| 18-40478429_G | 0.219048 | 210 | 0.304348 | 138 | 0.348101 | 158 | 0.441667 | 120 |
| 18-40807803_G | 0.435185 | 216 | 0.485714 | 140 | 0.613924 | 158 | 0.675 | 120 |
| 18-42386473_C | 0.268519 | 216 | 0.253623 | 138 | 0.443038 | 158 | 0.416667 | 120 |
| 18-44859933_G | 0.398148 | 216 | 0.342857 | 140 | 0.126582 | 158 | 0.083333 | 120 |
| 18-46490552_G | 0.388889 | 216 | 0.285714 | 140 | 0.5 | 158 | 0.466667 | 120 |
| 18-75187666_C | 0.149533 | 214 | 0.178571 | 140 | 0.170886 | 158 | 0.166667 | 120 |
| 18-75504584_G | 0.5 | 214 | 0.5 | 138 | 0.5 | 158 | 0.5 | 120 |
| 18-75780078_A | 0.406542 | 214 | 0.357143 | 140 | 0.259494 | 158 | 0.308333 | 120 |
| 18-75969689_A | 0.148148 | 216 | 0.173913 | 138 | 0.177215 | 158 | 0.208333 | 120 |
| 18-75992716_T | 0.365741 | 216 | 0.342857 | 140 | 0.234177 | 158 | 0.241667 | 120 |
| 18-76006633_G | 0.359813 | 214 | 0.352113 | 142 | 0.234177 | 158 | 0.241667 | 120 |
| 18-77703956_G | 0.14486 | 214 | 0.188406 | 138 | 0.170886 | 158 | 0.175 | 120 |
| 18-77725062_T | 0.135514 | 214 | 0.178571 | 140 | 0.164557 | 158 | 0.158333 | 120 |
| 18-77894560_G | 0.347222 | 216 | 0.342857 | 140 | 0.518987 | 158 | 0.558333 | 120 |
| 21-4512138_A | 0.475 | 200 | 0.401515 | 132 | 0.429577 | 142 | 0.415094 | 106 |
| 21-4515908_A | 0.256881 | 218 | 0.282609 | 138 | 0.202532 | 158 | 0.2 | 120 |
| 21-4598516_A | 0.429245 | 212 | 0.492754 | 138 | 0.506329 | 158 | 0.516667 | 120 |
| 21-4707138_T | 0.420561 | 214 | 0.464789 | 142 | 0.442857 | 140 | 0.488889 | 90 |
| 21-4800528_G | 0.264286 | 140 | 0.163043 | 92 | 0 | 78 | 0 | 26 |
| 21-4800562_G | 0.486111 | 216 | 0.387324 | 142 | 0.417722 | 158 | 0.433333 | 120 |
| 21-4898282_A | 0.360825 | 194 | 0.428571 | 126 | 0.359155 | 142 | 0.284314 | 102 |
| 21-6300122_G | 0.111111 | 90 | 0.043478 | 46 | Did not genotype successfully | | | |
| 21-6590487_A | 0.416667 | 216 | 0.421429 | 140 | 0.396104 | 154 | 0.5 | 116 |
| 21-6611863_G | 0.055556 | 108 | 0.05 | 60 | 0.15 | 80 | 0.136364 | 44 |
| 21-7509248_G | 0.435185 | 216 | 0.442857 | 140 | 0.405063 | 158 | 0.353448 | 116 |
| 21-48664783_T | 0.377358 | 212 | 0.271429 | 140 | 0.381579 | 152 | 0.258621 | 116 |
| 21-49216451_A | 0.351485 | 202 | 0.278571 | 140 | 0.266667 | 150 | 0.25 | 112 |
| 21-49368721_A | 0.075472 | 212 | 0.05 | 140 | 0.039474 | 152 | 0.070175 | 114 |
| 21-49882816_A | 0.226415 | 212 | 0.34058 | 138 | 0.311688 | 154 | 0.37931 | 116 |
| 21-49950751_C | 0.425234 | 214 | 0.392857 | 140 | 0.329114 | 158 | 0.391667 | 120 |
| 21-50238955_T | 0.327586 | 174 | 0.358491 | 106 | 0.461538 | 156 | 0.441667 | 120 |
| 21-50250540_A | 0.331683 | 202 | 0.301471 | 136 | 0.340278 | 144 | 0.294118 | 102 |
| 21-50321052_C | 0.028037 | 214 | 0.021739 | 138 | 0.063291 | 158 | 0.033898 | 118 |
| 21-50348105_C | 0.212963 | 216 | 0.35 | 140 | 0.259494 | 158 | 0.3 | 120 |
| 21-50383063_G | 0.232673 | 202 | 0.352113 | 142 | 0.25641 | 156 | 0.3 | 120 |
| 21-51305453_T | 0.065421 | 214 | 0.028571 | 140 | 0.075949 | 158 | 0.066667 | 120 |
| 21-51325270_T | 0.014851 | 202 | 0.014493 | 138 | 0 | 154 | 0 | 116 |
| 21-51353146_G | 0.376238 | 202 | 0.478873 | 142 | 0.493506 | 154 | 0.543103 | 116 |
| 21-51402003_C | 0.14486 | 214 | 0.107143 | 140 | 0.158228 | 158 | 0.133333 | 120 |
| 21-51408645_C | 0.171429 | 210 | 0.107143 | 140 | 0.064935 | 154 | 0.068966 | 116 |
| 21-51448245_A | 0.22449 | 196 | 0.234848 | 132 | 0.222222 | 144 | 0.166667 | 108 |
| 21-52365784_T | 0.134259 | 216 | 0.085714 | 140 | 0 | 158 | 0 | 120 |
| 21-52586460_0 | 0 | 218 | 0 | 142 | 0 | 158 | 0 | 118 |
| 21-52594185_G | 0.438679 | 212 | 0.371429 | 140 | 0.428571 | 154 | 0.362069 | 116 |
| 21-53288223_C | 0.064356 | 202 | 0.051471 | 136 | 0.160256 | 156 | 0.15 | 120 |
| 21-53443537_T | 0.238318 | 214 | 0.378571 | 140 | 0.259494 | 158 | 0.316667 | 120 |
| 21-53591449_C | 0.438679 | 212 | 0.384058 | 138 | 0.220779 | 154 | 0.189655 | 116 |
| 21-53794214_T | 0.453271 | 214 | 0.384058 | 138 | 0.208861 | 158 | 0.186441 | 118 |
| 21-53928489_G | 0.171296 | 216 | 0.137681 | 138 | 0.25641 | 156 | 0.183333 | 120 |
